# Clustering and percolation in protein loop structures

**DOI:** 10.1186/s12900-015-0049-x

**Published:** 2015-10-29

**Authors:** Xubiao Peng, Jianfeng He, Antti J. Niemi

**Affiliations:** Department of Physics and Astronomy, Uppsala University, P.O. Box 803, Uppsala, S-75108 Sweden; School of Physics, Beijing Institute of Technology, Beijing, 100081 People’s Republic of China; Laboratoire de Mathematiques et Physique Theorique CNRS UMR 6083, Fédération Denis Poisson, Université de Tours, Parc de Grandmont, Tours, F37200 France

**Keywords:** Loop modeling, Protein backbone, C *α* trace problem

## Abstract

**Background:**

High precision protein loop modelling remains a challenge, both in template based and template independent approaches to protein structure prediction.

**Method:**

We introduce the concepts of protein loop clustering and percolation, to develop a quantitative approach to systematically classify the modular building blocks of loops in crystallographic folded proteins. These fragments are all different parameterisations of a unique kink solution to a generalised discrete nonlinear Schrödinger (DNLS) equation. Accordingly, the fragments are also local energy minima of the ensuing energy function.

**Results:**

We show how the loop fragments cover practically all ultrahigh resolution crystallographic protein structures in Protein Data Bank (PDB), with a 0.2 Ångström root-mean-square (RMS) precision. We find that no more than 12 different loop fragments are needed, to describe around 38 % of ultrahigh resolution loops in PDB. But there is also a large number of loop fragments that are either unique, or very rare, and examples of unique fragments are found even in the structure of a myoglobin.

**Conclusions:**

Protein loops are built in a modular fashion. The loops are composed of fragments that can be modelled by the kink of the DNLS equation. The majority of loop fragments are also common, which are shared by many proteins. These common fragments are probably important for supporting the overall protein conformation. But there are also several fragments that are either unique to a given protein, or very rare. Such fragments are probably related to the function of the protein. Furthermore, we have found that the amino acid sequence does not determine the structure in a unique fashion. There are many examples of loop fragments with an identical amino acid sequence, but with a very different structure.

**Electronic supplementary material:**

The online version of this article (doi:10.1186/s12900-015-0049-x) contains supplementary material, which is available to authorized users.

## Background

Protein taxonomy [1–5] reveals that crystallographic protein structures have surprisingly little conformational diversity. It might be that the majority of different conformations have already been found [6, 7]. This apparent convergence in protein structure provides the rationale for the development of comparative modelling or threading techniques [8–12]. These approaches try to predict the tertiary structure of a folded protein using libraries of known protein structures as templates. According to the community-wide Critical Assessment for Structural Prediction (CASP) tests [13], at the moment this kind of methods have the best predictive power to determine a folded conformation.

In the loop regions, comparative modelling approaches still continue lacking in their precision [14, 15]. It is not uncommon that there are gaps in the loop regions that need to be filled by various insertion techniques. The success in loop modelling is also often limited to super-secondary structures where *α*-helices and *β*-strands are connected to each other by relatively short twists and turns [16, 17]. In the case of a very short loop, with no more than three residues, the shape can be determined by a combination of geometrical considerations and stereochemical constraints [18]. In the case of longer loops, both template based and template independent methods are being developed to predict their shapes [19–21]. The underlying assumption is that the number of loop conformations which can be accommodated by a given sequence should be limited. The different fragments which are already available in the Protein Data Bank (PDB) [22] database could then be used like *Lego bricks*, as structural building blocks in constructing the loops. A given amino acid sequence is simply divided into short fragments, and the shape of the ensuing loop is deduced using homologically related fragments that have known structures. The entire protein is then assembled by joining these fragments together. For the process of joining the fragments, both all-atom energy functions and comparisons with closely homologous template structures in the Protein Data Bank can be utilised [8, 9, 12, 14].

In the present article we propose a new systematic, purely quantitative method to identify and classify the modular building blocks of PDB loops; we identify a loop following the DSSP [23] convention. Our approach is based on a first-principles energy function [24–29]. It is built on the concept of *universality* [30–36] to model the fragments of even long protein loops in terms of different parameterisations of a unique *kink* that solves a variant [37, 38] of the discrete nonlinear Schrödinger (DNLS) equation [39, 40]. Our starting point is the observation made in [41] that over 92 % of loops in those PDB structures that have been measured with better than 2.0 Å resolution, can be composed from 200 different parameterisations of the kink profile, with better than 0.65 Ångström RMSD (root-mean-square-distance) accuracy. Here we refine this observation, with the aim to develop it into a systematic loop fragment classification scheme. For this we consider only those ultrahigh precision PDB structures that have been measured with better than 1.0 Å resolution. This ensures that the B-factors in the loop regions are small, and in particular that the structures have not been subjected to extensive refinement procedures. Indeed, two loop fragments should be considered different only, when the average interatomic distance is larger than the average Debye-Waller B-factor fluctuation distance. If the B-factors are large, any systematic attempt to identify and/or distinguish two fragments becomes ambiguous. In the case of these intra-high resolution structures we can aim for the RMSD precision of 0.2 Å. We estimate this to be the extent of zero point fluctuations *i.e.* a distance around 0.2 Å corresponds to the *intrinsic* uncertainty in the determination of heavy atom positions along the protein backbone. Thus any difference less than 0.2 Å between average atomic coordinates is essentially *undetectable*. By explicit constructions, we show how in the case of this subset of ultrahigh resolution PDB protein structures, the loops can be systematically modeled using combinations of the unique kink of the generalised DNLS equation. As such, our approach provides a foundation for a general approach to classify loops in high precision crystallographic PDB structures, in terms of an energy function based first-principles mathematical concept.

## Method

### C *α* based Frenet frames

**Fig. 1:**
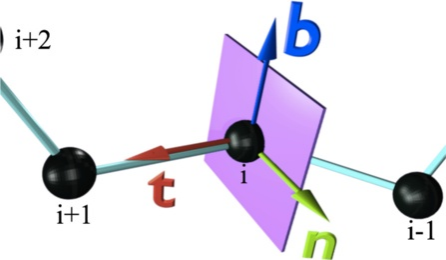
Discrete Frenet frame. (Color online) Discrete Frenet frame vectors **t**,**n**,**b** are shown in arrows

Let **r**_*i*_ (*i*=1,…,*N*) be the coordinates of the protein backbone *α*-carbon (C *α*) atoms. The indexing starts from the N terminus. At each **r**_*i*_ we introduce the discrete Frenet frame (**t**_*i*_,**n**_*i*_,**b**_*i*_) shown in Fig. 1 following the method in reference [42].

**Fig. 2:**
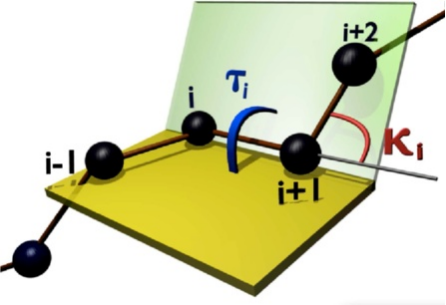
Bond and torsion angles. (Color online) Bond (*κ*_*i*_) and torsion (*τ*_*i*_) angles with the definitions as Eqs. (1) and (2) are noted in the figure

From the Frenet frames, we define the virtual C *α* backbone bond (*κ*) and torsion (*τ*) angles shown in Fig. 2 as follows,

(1) $$ \cos\kappa_{i+1} \ = \ \mathbf t_{i+1} \cdot \mathbf t_{i}   $$

(2) $$ \cos\tau_{i+1} \ = \ \mathbf b_{i+1} \cdot \mathbf b_{i}   $$

**Fig. 3:**
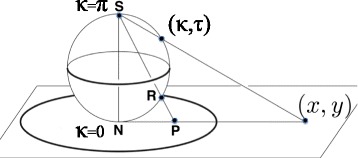
Stereogrphic projection. (Color online) Stereographic projection of two sphere with latitude *κ* and longitude *τ*

We identify the bond angle *κ*∈ [ 0,*π*] with the latitude angle of a two-sphere which is centered at the C *α* carbon; the tangent vector **t** points towards the north-pole where *κ*=0. The torsion angle *τ*∈ [ −*π*,*π*) is the longitudinal angle on the sphere. We have *τ*=0 on the great circle that passes both through the north pole and through the tip of the normal vector **n**, and the longitude increases in the counterclockwise direction around the tangent vector. We stereographically project the sphere onto the complex (*x*,*y*) plane from the south-pole

(3) $$ z=x+iy \ \equiv \ \sqrt{x^{2} + y^{2}} \, e^{i\tau} \ = \ \tan\left(\kappa/2 \right) \, e^{i\tau}   $$

as shown in Fig. 3; the north-pole where *κ*=0 becomes mapped to the origin (*x*,*y*) =(0,0) while the south-pole *κ*=*π* is sent to infinity.

We often find it convenient to extend the range of the latitude *κ* from positive to arbitrary real values. We compensate for this double covering of the sphere by introducing the following discrete $\mathbb Z_{2}$ gauge transformation

(4) $$ \begin{array}{llll} \ \ \ \ \ \ \ \ \ \kappa_{k} & \to & - \ \kappa_{k} & \quad\text{for \ \ all} \ \ k \geq i \\ \ \ \ \ \ \ \ \ \ \tau_{i} & \to & \quad\tau_{i} - \pi \end{array}   $$

This transformation has no effect on the backbone coordinates **r**_*i*_, and it leaves the C *α* backbone intact.

### The C *α* trace visualization, loops and kinks

#### The C *α* map

**Fig. 4:**
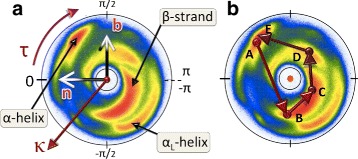
C _*α*_ stereographical projection map and folding index. (Color online) **a** The stereographically projected Frenet frame map of backbone C *α* atoms, with major secondary structures identified. Also shown is the directions of the Frenet frame normal vector **n**; the vector **t** points upwards and colour coding corresponds to the number of PDB entries with red large, blue small and white none. **b** An example of a loop (kink) trajectory, starting (*a*) and ending (*e*) in *α*-helical position

We visualise the backbone C *α* trace of a given protein in terms of a trajectory on the stereographically projected two-sphere, as follows [43–45]: At the location of each C *α* we introduce the corresponding discrete Frenet frames (**t**_*i*_,**n**_*i*_,**b**_*i*_). The base of the *i*^*th*^ tangent vector **t**_*i*_ is located at the position **r**_*i*_ of the *i*^*th*^ C *α* carbon, it coincides with the centre of the two-sphere and the vector **t**_*i*_ points towards the north-pole. We translate the sphere from the location of the *i*^*th*^ C *α* to the location of the (*i*+1)^*th*^ C *α*, without introducing any rotation of the sphere with respect to the *i*^*th*^ Frenet frames. We identify the direction of **t**_*i*+1_, *i.e.* the direction towards the C *α* carbon at site **r**_*i*+2_ from the site **r**_*i*+1_, on the surface of the sphere in terms of the ensuing spherical coordinates (*κ*_*i*_,*τ*_*i*_). We repeat the procedure for all the backbones in PDB. To enhance statistics, for visualisation purposes we use here those protein structures that have been measured with better than 2.0 Å resolution, which gives us the map shown in Fig. 4a; see also Figure S1 in Additional file 1. The color intensity correlates directly with the statistical distribution of the (*κ*_*i*_,*τ*_*i*_): red is large, blue is small and white is none. The map describes the direction of the C *α* carbon at **r**_*i*+2_ as it is seen at the vertex **r**_*i*+1_, in terms of the Frenet frames at **r**_*i*_.

Note how the statistical distribution in Fig. 4 concentrates within an annulus, roughly between the latitude angle values (in radians) *κ*∼1 and *κ*∼*π*/2. The exterior of the annulus is a sterically excluded region. The entire interior is in principle sterically allowed, but it is very rarely occupied in the case of folded proteins. The four major secondary structure regions, *α*-helices, *β*-strands, left-handed *α*-helices and loops, are identified according to their PDB classification. For example, (*κ*,*τ*) values (in radians) for which

(5) $$ \left\{ \begin{array}{lll} \kappa_{i} & \approx & \frac{\pi}{2} \\ \tau_{i} & \approx & 1 \end{array} \right.   $$

describes a right-handed *α*-helix, and values for which

(6) $$ \left\{ \begin{array}{lll} \kappa_{i} & \approx & 1 \\ \tau_{i} & \approx & \pm \pi \end{array} \right.   $$

describes a *β*-strand. We note that the Fig. 4a is akin the Newman projection of stereochemistry: The vector **t**_*i*_ which is denoted by the red dot at the center of the figure, points along the backbone from the proximal C *α* at **r**_*i*_ towards the distal C *α* at **r**_*i*+1_, and the colour intensity displays the statistical distribution of the **r**_*i*+2_ direction. We also note that the Fig. 4 provides non-local information on the backbone geometry; the information content extends over several peptide units. This is unlike the Ramachandran map, which can only provide localised information in the immediate vicinity of a single C *α* carbon. As shown in [46], the C *α* backbone bond and torsion angles (*κ*_*i*_,*τ*_*i*_) are sufficient to reconstruct the entire backbone, while the Ramachandran angles are not.

In Fig. 4b we visualise as an example a path made by a generic protein loop that connects two right-handed *α*-helical structures. A notable property of the trajectory drawn in Fig. 4b is that it encircles the north-pole of the two-sphere. It turns out that this kind of encircling is quite generic for loops, even entire folded proteins [47]. Consequently, we assign to each loop a winding number which we term *folding index* that we denote *Ind*_*f*_ [47] and define as follows,

(7) $$\begin{array}{*{20}l} {Ind}_{f}&=&\left[\frac{\Gamma}{\pi}\right]  \end{array} $$

(8) $$\begin{array}{*{20}l} \Gamma&=&\sum\limits_{i=n_{1}+2}^{n_{2}-2} \left\{ \begin{array}{ll} \tau_{i}-\tau_{i-1}-2\pi & ~~\text{if}~~ \tau_{i}-\tau_{i-1} > \pi\\ \tau_{i}-\tau_{i-1}+2\pi & ~~\text{if}~~ \tau_{i}-\tau_{i-1} < -\pi\\ \tau_{i}-\tau_{i-1} & ~~\text{otherwise} \end{array}\right.  \end{array} $$

Here [*x*] denotes the integer part of *x*, and *Γ* is the total rotation angle (in radians) that the projections of the C *α* atoms of the consecutive loop residues make around the north pole. The folding index is a positive integer when the rotation is counterclockwise, and a negative integer when the rotation is clockwise. The folding index can be used to detect and classify loop structures and entire folded proteins, in terms of its values. The value is equal to twice the number of times the ensuing pathway encircles the north-pole in the map of Fig. 4; for the trajectory shown in Fig. 4b the folding index is +2.

### The discrete nonlinear Schrödinger equation

The virtual bond length between two neighboring C *α* atoms is essentially constant, with the value 3.8 Å. Accordingly the Helmholtz free energy for the C *α* trace backbone can be expressed in terms of the virtual bond angles *κ*_*i*_ and dihedral angles *τ*_*i*_ only. To the leading order in the infrared limit the result coincides with

(9) $$ \begin{aligned} F &= - \sum\limits_{i=1}^{N-1} 2\, \kappa_{i+1} \kappa_{i} + \sum\limits_{i=1}^{N} \left\{2 {\kappa_{i}^{2}} + c \, \left({\kappa_{i}^{2}} - m^{2}\right)^{2}\right.\\ &\quad\left.+\, b \, {\kappa_{i}^{2}} {\tau_{i}^{2}} + d \, \tau_{i} + e \, {\tau^{2}_{i}} + q \, {\kappa_{i}^{2}} \tau_{i} \vphantom{\left\{2 {\kappa_{i}^{2}} + c \, \left({\kappa_{i}^{2}} - m^{2}\right)^{2}\right.}\right\}  \end{aligned}  $$

This is essentially the Hamiltonian of the discrete nonlinear Schrödinger equation [39, 40]; for a detailed derivation we refer to [24–29]. Remarkably, the free energy (9) supports a kink (topological soliton) as a classical solution [37, 38]. An *excellent* approximation of a kink can be obtained by *naively* discretising the kink solution of the *continuum* nonlinear Schrödinger equation [37, 38, 48]

(10) $$ \kappa_{i} = \frac{\mu_{1} \exp\left[ \sigma_{1} (i-s) \right] + \, \mu_{2} \exp\left[ - \sigma_{2} (i-s)\right]} {\exp\left[ \sigma_{1} (i-s) \right] + \exp\left[- \sigma_{2} (i-s)\right]}   $$

The torsion angles *τ* are then expressed as functions of the bond angles *κ*

(11) $$ \tau_{i} [\!\kappa] \ = \ - \frac{1}{2} \, \frac{d + q {\kappa_{i}^{2}}}{e + b{\kappa_{i}^{2}}}   $$

For the torsion angles, from (11) we conclude that the overall scale of the parameters (*d*,*q*) and (*e*,*b*) cancel in the expression (11). This leaves us with only three independent parameters. In (10) there are four parameters when we use translation invariance to remove *s*. Thus the profile of a single kink becomes *fully* determined in terms of *seven* independent parameters. This coincides *exactly* with the number of independent coordinates along a C *α* backbone segment, with *six* residues. For this, we may always place the first residue to coincide with the origin of a Cartesian (*xyz*) coordinate system. We can always place the second residue along the *z*-axis, and we can always place the third residue on the *x*=0 plane. Thus, there is only one independent coordinate for the three first residues. Since the remaining three residues can each be placed to arbitrary angular directions, there are six additional independent coordinates. Accordingly, a segment with six residues indeed engages seven independent parameters.

### Clustering and percolation

We shall classify the loop structures in PDB in terms of the following *clustering algorithm*:

- We define a *cluster* to be a set of loop fragments such that for each fragment in a given cluster there is at least one other fragment within a prescribed RMS cut-off distance.
  Two clusters are disjoint, when the RMSD between any fragment in the first cluster and any fragment in the second cluster exceeds this prescribed RMS cut-off distance.
- We define the *initiator* of a cluster to be an *a priori* random loop fragment which defines the cluster by *completion*, as follows: We start with the initiator. We identify all those fragments in our entire data set which deviate from the initiator by less than the given RMS cut-off distance. We continue the process by identifying all those fragments, that deviate from the fragments that we have identified in the previous step, by less than the RMS cut-off distance. We repeat the procedure until we find no additional fragments in PDB, within the RMS cut-off distance from *any* of those fragments that have been identified in the previous steps.

The cluster is clearly independent of its initiator, any element of the cluster could be used as the initiator. But the cluster depends on the RMS cut-off distance. Moreover, if the RMS cut-off distance is too large, no clear clustering is observed.

According to [49] for a PDB protein structure which is measured with resolution 2.0 Å or better, the characteristic values of the thermal B-factors are mostly less than around

(12) $$ B_{max} \ \buildrel < \over \sim \ 35 \ \text{\r{A}}^{2}   $$

From the Debye-Waller relation we then obtain the following estimate for the one standard deviation error in the atomic coordinates

(13) $$ \sqrt{<x^{2} >}_{max} \ = \ \sqrt{\frac{B_{max}}{8\pi^{2}}} \ \approx 0.65 { \text{\r{A}}}   $$

Thus, two loop fragments that have been measured with 2.0 Å resolution should be (in average) considered different only, when their RMS distance exceeds 0.65 Å.

**Fig. 5:**
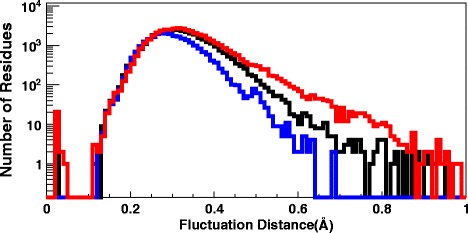
Debye-Waller fluctuations for PDB structures. Number of C *α* entries in PDB measured with resolution under 1.0 Å *vs.* the Debye- Waller fluctuation distance. The blue line denotes the Debye-Waller fluctuation distance distribution for *β*-sheets, black for *α*-helices, and red for loop. The entries near 0 correspond to the PDB structures 1ETL,1ETM and 1ETN. Note the logarithmic scale

The construction of PDB loop fragments in terms of the kink profile (10), (11) in those crystallographic protein structures which have been measured with resolution 2.0 Å or better, has been addressed in [41]. There, it was found that over 92 percent of loops can be covered in a modular fashion by 200 explicit kink profiles (10), (11), with RMSD accuracy that matches (13) *i.e.* with less than 0.65 Å RMSD deviation from the crystallographic structure. Thus 0.65 Å RMS distance is the appropriate RMS cut-off value, to search for for the more refined clustering patterns in those crystallographic structures which have been measured with resolution 2.0 Å. However, we find that the value 0.65 Å is too large, to observe clear clustering patterns. Accordingly, we shall search for clustering by considering only those PDB structures that have been determined with the ultrahigh resolution 1.0 Å or better. For these ultrahigh resolution structures, a precision better than the value (13) can be expected. To determine an appropriate value, we display in Fig. 5 the number of all C *α* atoms in all currently available PDB structures, that have been measured with resolution 1.0 Å or better, as a function of their Debye-Waller fluctuation distance. For most of the structures, the fluctuation distance is clearly below the upper bound (13); the maximum of the curve is located at around 0.3 Å. We also observe the (essential) absence of structures with a fluctuation distance less than 0.1 Å; historically this distance is considered as the boundary wavelength between x-rays and *γ*-rays.

Using a combination of Fig. 5 with various tests that we have performed, we have arrived at the conclusion that 0.2 Å in RMS distance can be *currently* adopted as a reasonable estimate for the minimal zero-point fluctuation distance in ultra-high resolution structures, those that have been measured with better than 1.0 Å resolution. Thus we shall try and see, to what extent loops in these protein structures can be classified in terms of elemental fragments, such that two fragments are considered different when their RMS distance exceeds 0.2 Å. According to Fig. 5, over 99 % of individual C *α* carbons that have been measured with below 1.0 Å resolution, have a B-factor fluctuation distance which is larger than 0.2 Å; our choice of cut-off distance is close to the 3- *σ* level.

We note that other cut-off values can be introduced; the ultimate appears to be 0.1 Å. But our qualitative conclusions are fairly independent of the value chosen, provided it is small enough to provide a clustering pattern. In this article our goal is to present a proof-of-concept. To our knowledge, no related analysis has been previously attempted, to systematically classify the loop structures in ultra-high resolution crystallographic protein conformations, in a quantitative fashion using an energy function. In particular, no commonly accepted experimental standard exist, that we could rely on, to infer the “most preferred” cut-off value. We hope that such a value can be eventually inferred, from careful experimental measurements. Thus, at the moment we have no criterion to prefer any other particular value, 0.2 Å *i.e.* around 3- *σ* appears to be a reasonable choice at this point.

We start the identification of loop fragments, using the set of 200 fragments constructed in [41]. But our results are independent of the starting point, quite similar results can be obtained using a fairly generic set of loop fragments as a starting point. We note that the fragments in [41] have between five and nine residues, and most of them (116 out of 200) have six residues. We have already argued that six is the optimal number of residues in a loop fragment, as it matches the number of independent parameters in the kink profile (10), (11). Thus, we shall consider only fragments that have six residues, in the clustering algorithm. In this manner, we find that we can classify *all* PDB fragments into clusters, each determined by their initiator.

We have found that there are clusters that have a very large number of fragments. But we also find that there are clusters with only a single, or very few fragments. It is natural to expect that those clusters which are large, contain mostly fragments that are *structurally* important. On the other hand, those clusters which are small should include mainly fragments that are *functionally* important. Furthermore, we find several examples of amino acid sequences that are included in different clusters: The sequence does not define the structure, in a unique fashion. This leads us to address the concept of *cluster percolation*: Given the sequence of a loop fragment in a cluster, percolation means that there are other, possibly new clusters where the same sequence appears but with a different structure.

## Results

### Clustering

We have constructed our clusters by starting with the 200 loop fragments that were introduced in [41]. Around 92 % of all loops in those PDB structures that have been measured with resolution better than 2.0 Å, are within a 0.65 Å RMS distance from some of the 200 loop fragments. However, when we decrease the RMSD cut-off distance to 0.2 Å, which is the cut-off distance used in the present article, the coverage drops to below 2 % [41].

We remark that the authors of reference [41] did not investigate *clustering*, as the concept is defined here. In [41] all the RMS distances were evaluated from the *fixed* set of 200 loop fragments, and the coverage of PDB loop structures was determined in terms of these fixed loop fragments.

When we specify to the present subset of PDB structures in [41] that have been measured with better than 1.0 Å resolution, we find that a total of 102 out of the 200 fragments in [41] have been measured with this resolution. We use these 102 loop fragments as the initiators, to start our clustering construction.

#### clusters

The 102 loop fragments in [41] that have been measured with better than 1.0 Å resolution, have between five and nine residues. Here we have argued that a loop fragment modelled by (10), (11) has six residues. There are 70 such clusters among the 200, but only 14 of them contain more than 30 fragments. Moreover, two of these merge together into an *α*-helical structure, when we subject them to our clustering algorithm; we call them *bends* instead of kinks. The remaining 12 loop fragments determine clusters which cover around 38 % of the 1.0 Å protein loop structures, when we use our 0.2 Å RMSD cut-off. These loop fragments are our final initiators. In Table 1 we list the PDB entry codes and residue numbers of these initiators.

**Table 1:** The list of 12 initiators for clusters that have 6 residues and give rise to 30 or more entries in the ensuing clusters (PDB code, chain, PDB sites), together with the number of entries

Cluster *#*	Initiator	*#* entries
I	1vyr_A (174–179)	76
II	1g4i_A (56–61)	138
III	1gkm_A (163–168)	186
IV	4f18_A (1244–1249)	199
V	1a6m_A (18–23)	215
VI	1cex_A (140–145)	273
VII	1a6m_A (56–61)	308
VIII	1iee_A (47–52)	481
IX	1brf A (5–10)	1166
X	1ixh_A (200–205)	1405
XI	2o7a_A (62–67)	1586
XII	1gkm_A (9–14)	2374

We proceeded to describe some of the major features of the ensuing 12 clusters. Additional details including a breakdown according to amino acid constituents in each cluster, are presented in Figure S2 of Additional file 1.

**Fig. 6:**
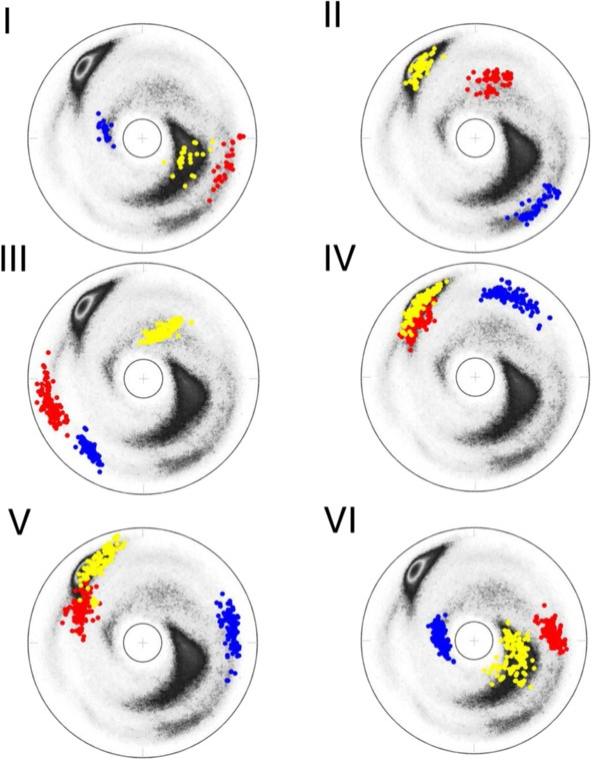
The stereographic maps of 12 clusters *I-VI*. The clusters I-VI in Table 1 are shown on the stereographic map like Fig. 4 a; In each panel the order along the C *α* backbone is *r* *e* *d* → *b* *l* *u* *e* → *y* *e* *l* *l* *o* *w*

**Fig. 7:**
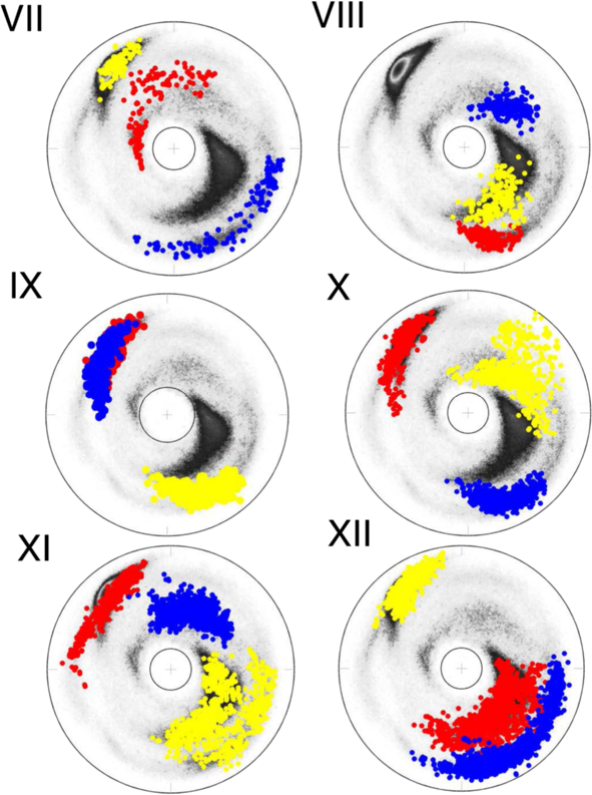
The stereographic maps of 12 clusters *VII-XII*. The clusters VII-XII in Table 1 are shown on the stereographic map like Fig. 4 a; The ordering along the C *α* backbone is *r* *e* *d* → *b* *l* *u* *e* → *y* *e* *l* *l* *o* *w*

The Figs. 6 and 7 show the (*κ*,*τ*) distribution in each of the 12 clusters on the stereographically projected two-sphere of Fig. 4; note that the definition of bond angle takes three residues while the definition of torsion angle takes four. Thus for a 6 residue loop fragment there are three (*κ*,*τ*) pairs. The fourth *κ*-value could be used to refine the loop classification, but here this possibility is not considered.

**Fig. 8:**
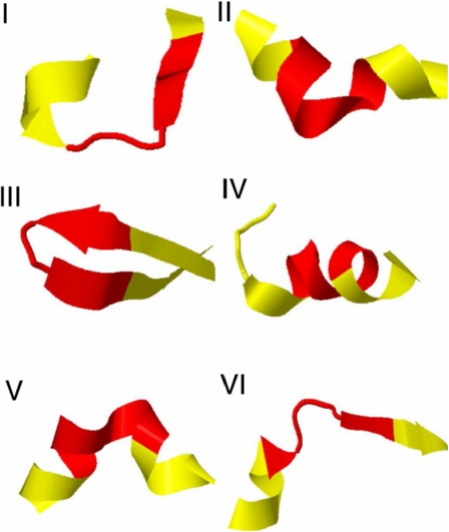
The initiators of the 12 clusters *I-VI*. The initiators I-VI listed in Table 1 are shown in their three dimensional backbone environment. The *(dark) red color* identifies the initiator, and the *(light) yellow color* identifies the immediate backbone environment

**Fig. 9:**
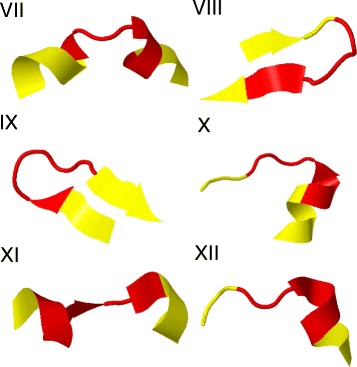
The initiators of the 12 clusters *VII-XII*. The initiators *VII-XII* listed in Table 1 are shown in their three dimensional backbone environment. The *(dark) red color* identifies the initiator, and the *(light) yellow color* identifies the immediate backbone environment

In Figs. 8 and 9 we show the three dimensional pictures of the initiators of the twelve clusters.

**Fig. 10:**
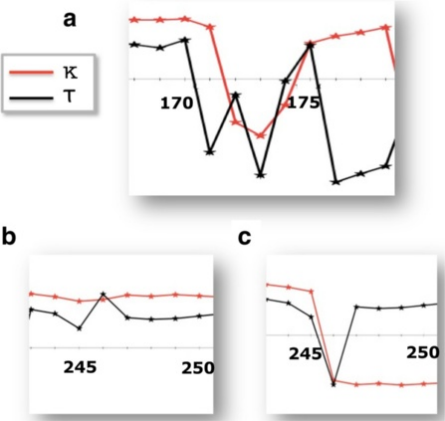
The (*κ*,*τ*) spectrum of initiator I and IV. The figure **a** shows the $\mathbb Z_{2}$ gauge transformed spectrum of bond and torsion angles in the case of the initiator I. This reveals that the initiator is a two-kink configuration that forms a loop between *α*-helical and *β*-stranded regular secondary structures. The figures **b** and **c** show the bond and torsion angle spectra of the bend-like initiator IV prior and subsequent to the $\mathbb Z_{2}$ gauge transformation, respectively

A detailed inspection reveals that except for IV, all the initiators have the canonical structure of a single kink, in terms of the folding index (8). Moreover, the initiator I is part of a short loop connecting an *α*-helix and a *β*-strand. However, the bond and torsion angle spectrum which we display in Fig. 10a shows that this loop is actually a pair of two kinks which are very close to each other, and the initiator I is the second kink along the backbone.

On the other hand, a comparison with (8) suggests that the initiator IV exhibits a somewhat small variation in the values of the torsion angles, for a kink. This can be seen in Fig. 6. The torsion angle values suggest that the initiator IV resembles more a bent *α*-helix than a kink. In Fig. 10b, c we show the spectrum of the bond and torsion angles of the initiator IV, both before and after we have implemented the $\mathbb Z_{2}$ gauge transformation. Since this bent structure determines an isolated cluster according to our 0.2 Å cut-off criteria, it is included among our loop fragments.

**Fig. 11:**
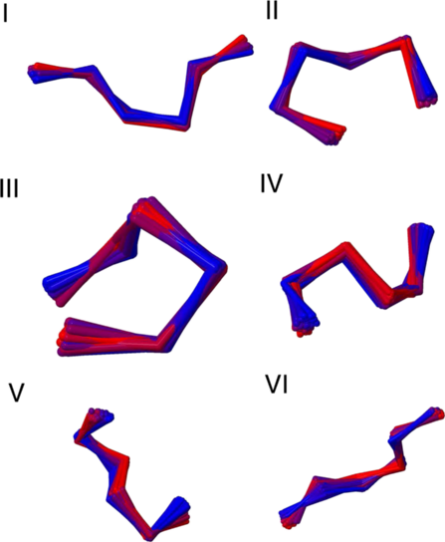
The 3D superimposed structures for 12 clusters *I-VI*. The clusters I-VI in Table 1 are superimposed in three dimensions. The colour ranges from *red* (initiator) to *blue* (the entry with largest RMSD distance from initiator)

**Fig. 12:**
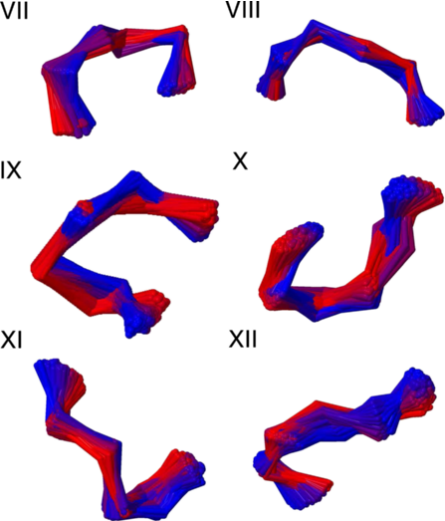
The 3D superimposed structures for 12 clusters *VII-XII*. The clusters VII-XII in Table 1 are superimposed in three dimensions. The colour ranges from *red* (initiator) to *blue* (the entry with largest RMSD distance from initiator)

In Figs. 11 and 12 we show the three dimensional figures of each of the twelve clusters, including all the entries.

Finally, we have also investigated how the coverage of the 12 clusters increases, when we increase the cut-off distance. The results are shown in Table 2.

**Table 2:** The coverage of the 12 clusters obtained using the initiators in Table 1, as a function of the cut-off distance

Cut-off (Å)	0.2	0.3	0.4	0.5
Coverage (%)	37.8	43.6	49.6	56.4

#### Cluster elongation and completion

**Fig. 13:**
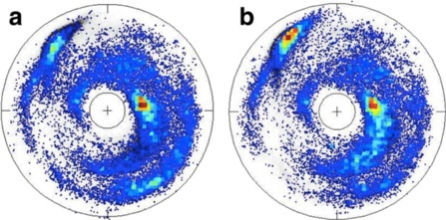
The stereographic map generated by cluster 1p1x_A (80–84). In **a** the distribution of the first (*κ*,*τ*) and in **b** the distribution of the second (*κ*,*τ*). Note the widely spreaded distributions of this cluster

In addition of the 12 initiators listed in Table 1, among the 102 loop fragments of [41] that we have considered, there is also one initiator that has only five residues. The PDB code is 1p1x_A (80–84). The ensuing cluster with five residue long elements is very large: There are a total of 42618 entries. The reason for the occurrence of such a large cluster is that the RMSD clustering criteria 0.2 Å is too large for revealing clustering patterns in five-residue-long loop segment: The five-residue-long loop fragment covers all the five-residue-long loops, within the chosen cut-off criterion. In Fig. 13 we show the distribution of (*κ*,*τ*) values in this cluster.

There is also an overlap with each of the 12 clusters that we obtained previously. Together the 13 clusters cover around 96.1 % of all PDB loop structures.

It is apparent that an initiator with only five residues is too short to identify a clustering pattern of PDB loops, even with 0.2 Å precision. Consequently we have elongated this initiator. For this, we have systematically added residues at the beginning and at the end of the individual elements in its cluster, to search for clustering patterns. For example, we may take the element 1p1x_A (80–84), elongate it to 1p1x_A (80–85) and 1p1x_A (79–84), and then use these two elongated ones as initiators to do the clusterings: We denote by H, S and L a residue which is located in a helix, strand and loop respectively, according to the PDB classification. The five residue long cluster which is generated by 1p1x_A (80–84) consists of several different elements, such as for example LLLLL, HLLLL, LLLLS *etc*.

**Fig. 14:**
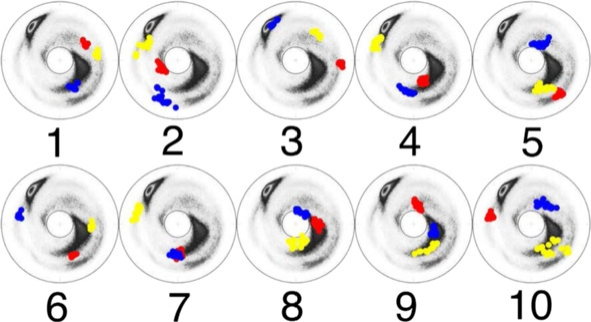
The stereographic map of the first 10 clusters in Table 3. The ordering along the C *α* backbone is *r* *e* *d* → *b* *l* *u* *e* → *y* *e* *l* *l* *o* *w*

**Fig. 15:**
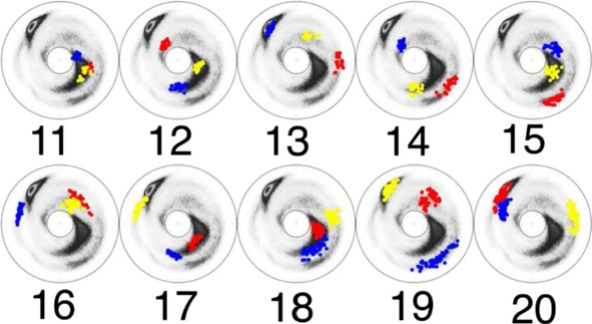
The stereographic map of the clusters **11**–**20** in Table 3. The ordering along the C *α* backbone is *r* *e* *d* → *b* *l* *u* *e* → *y* *e* *l* *l* *o* *w*

**Fig. 16:**
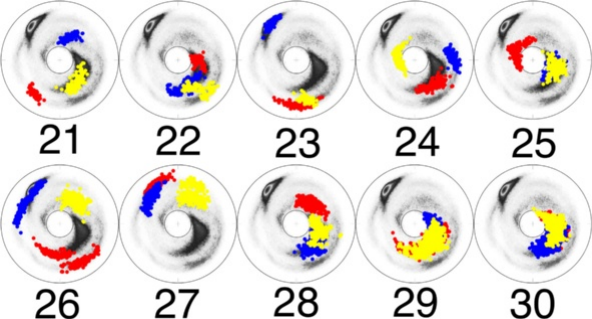
The stereographic map of the clusters **21**–**30** in Table 3. The ordering along the C *α* backbone is *r* *e* *d* → *b* *l* *u* *e* → *y* *e* *l* *l* *o* *w*

**Fig. 17:**
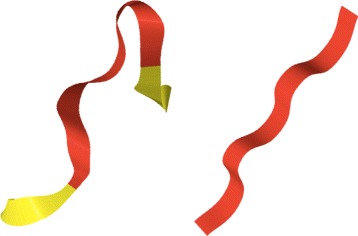
The initiators 29 (*left*) and 30 (*right*) in Table 3. The cluster 29 consists of loops, while the cluster 30 consist of regular *β*-strands

As an example, we have selected the pattern LLLLL which has the largest number of elements; there are a total of 7901 elements. We have elongated each of these 7901 elements into a protein loop fragment with six residues, by incorporating the corresponding PDB residue which is either right before the first L residue, or immediate after the last L residue. In this manner we find 15802 different loop fragments with six residues each. We have investigated the corresponding clustering patterns: There are 30 new clusters with more than 30 elements, bringing the total number of the clusters with more than 30 elements, to 42. We list these 30 additional clusters in Table 3. In Figs. 14, 15 and 16 we display the (*κ*,*τ*) distributions of these 30 clusters. A visual inspection of these clusters reveals, that at the level of the (*κ*,*τ*) distribution the cluster 26 appears to display additional sub-clustering. But the present cut-off value 0.2 Å is not sufficiently refined to detect this sub-clustering, at the level of RMS distance. Furthermore, the clusters 29 and 30 both appear to merge with the regular *β*-strand. In Fig. 17 we show the corresponding initiators: The cluster 29 is clearly a loop, while the cluster 30 consist of the regular *β*-strand and thus we exclude it from our set of loop fragments. This leaves us with a total of 41 clusters, with 30 or more loop fragments. These clusters cover around 52 % of all loop structures in PDB.

**Table 3:** The 30 clusters with six residues, obtained by elongation of the LLLLL subset of the cluster which is generated by 1p1x_A (80–84)

Cluster #	Initiator	Match #	Cluster #	Initiator	Match #
1	1kwf_A (324–329)	32	16	1xg0_A (15–20)	96
2	1byi_A (123–128)	34	17	2pve_A (23–28)	98
3	4iau_A (78–83)	34	18	1vyr_A (23–28)	114
4	2o9s_A (841–846)	37	19	1j0p_A (54–59)	135
5	4ayo_A (233–238)	37	20	2rh2_A (48–53)	151
6	1pwm_A (171–176)	38	21	3p8j_A (240–245)	200
7	1gdq_A (123–128)	39	22	4gda_B (62–67)	240
8	2wur_A (30–35)	40	23	7a3h_A (232–237)	309
9	3zsj_A (190–195)	41	24	1n55_A (31–36)	368
10	4kxu_A (257–262)	42	25	1f94_A (40–45)	507
11	1n4u_A (121–126)	43	26	2pfh_A (305–310)	628
12	1nls_A (155–160)	49	27	1ab1_A (41–46)	723
13	3dk9_A (356–361)	51	28	1gci_A (188–193)	777
14	1o7j_C (119–124)	52	29	3ne0_A (1094–1099)	1505
15	4hen_A (169–174)	95	30	3hyd_A (1–6)	2275

By completing the elongation process we have identified 3240 different clusters with 0.2 Å cut-off. These clusters cover around ∼85 % of all those PDB loop sites in our set of resolution better than 1.0 Å proteins. Among these clusters there are 1677 unique ones, in the sense that the cluster has only single element. Thus, around 14 % of all loop structures in PDB appear to be unique, to the given protein. In addition, there are 1531 *rare* clusters with two or more, but less than 32 elements. Thus, there are 32 clusters with 32 or more elements.

The remaining ∼15 % of loop fragments that are not covered by the 3240 clusters, can be constructed by completion. For example, we can search for novel clusters by using the patterns other than LLLLL in the five residue cluster generated by 1p1x_A (80–84). But when the four patterns HLLLL, LLLLH, SLLLL and LLLLS are included the coverage increases no more than around one per cent.

### Cluster percolation

We have also investigated the relation between the sequence and the structure, using the 42 clusters listed in Tables 1 and 3. Here we only describe some of the major features, more details can be found in Figure S3 in Additional file 1.

There are several examples of identical sequences that correspond to different structures in different proteins. Accordingly a sequence clearly does *not* determine a unique structure. When a given sequence gives rise to multiple structures, we have a phenomenon we call *cluster percolation*. These sequences with multiplet structures may be utilised to try and introduce novel clusters.

For example, in Table 4 those sequences that are found both in Cluster VIII and outside of it, are listed, together with their PDB identifications and RMSD to the initiator of Cluster VIII.

**Table 4:** Sequences that appear both in and outside of cluster VIII; only the entry outside of the cluster is identified. The RMSD is evaluated from the initiator of cluster VIII; H stands for helix, L for loop and S for strand

Sequence	PDB entry	PDB structure	RMSD(Å)
TDGSTD	2vb1_A (47–52)	LLLLSS	0.24
TDGSTD	3lzt_A (47–52)	LLLLSS	0.26
TDGSTD	4lzt_A (47–52)	LLLLSS	0.27
DAGMRF	3odv_A (20–25)	HHLLSS	0.71
ESGNVV	2agt_A (126–131)	LLLLLL	0.63
ESGNVV	2pzn_A (126–131)	LLLLLL	0.72
ESGNVV	3u2c_A (126–131)	LLLLLL	0.54
ADGKPV	4hen_A (54–59)	SLLSSS	1.43
ESGLSK	1g2y_B (18–23)	HHHLHH	1.19
NVGWPR	1mn8_B (47–52)	HLLLLL	0.79
KDGVAD	4a7u_A (91–96)	LLLLSS	0.68
SDGNGM	1iee_A (100–105)	HLLHHH	1.12
SDGNGM	2vb1_A (100–105)	HHLLHH	0.38
SDGNGM	4b4e_A (100–105)	HLLHHH	1.07
SDGNGM	4lzt_A (100–105)	HLLLLH	0.33
QQGLTL	3akq_A (161–166)	HHLLLL	0.62
QQGLTL	3akt_A (161–166)	HHLLLL	0.66
QQGLTL	3akt_B (161–166)	HHLLLL	0.59

**Fig. 18:**
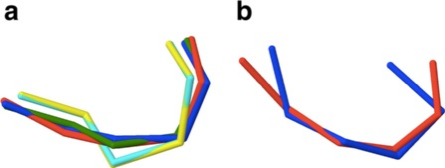
Examples of percolation in Cluster VIII, listed in Table 4. In **a** the SDGNGM entries 2vb1 A (100–105) (*blue*), 4lzt A (100–105) (*green*), 1iee A (100–105) (*yellow*) and 4b4e A (100–105) (*cyan*) with with the initiator 1iee A (47–52) (*red*). In **b** the ADGKPV entry 4hen A (54–59) (*blue*) with the initiator 1iee A (47–52) (*red*)

As an example, in Fig. 18a we compare the four PDB structures that have the identical sequence SDGNGM in the Table 4. The difference between the two mutually similar structures 2vb1 A (100–105) and 4lzt A (100–105) to the two equally mutually similar structures 1iee A (100–105) and 4b4e A (100–105) is visually apparent. A visual comparison with the Cluster VIII in Fig. 12 also reveals that both 1iee A (100–105) and 4b4e A (100–105) are clearly outside of this cluster.

Figure 18b shows the comparison of the sequence ADGKPV to the initiator. The difference between the structures of 4hen A (54–59) and the initiator is again clear. The structure of 4hen A (54–59) is also quite different from the structures in Fig. 18a, and from the Cluster VIII shown in Fig. 12.

**Fig. 19:**
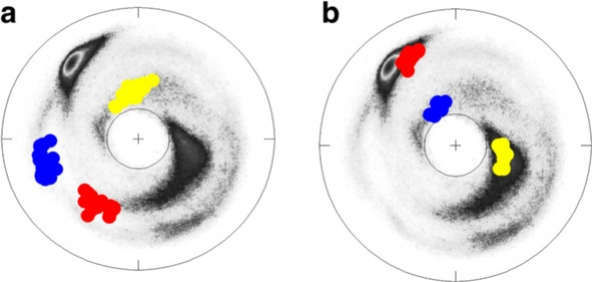
The (*κ*,*τ*) distributions of the two clusters with more than 30 entries obtained by percolation. Clusters with initiators **a** 1ix9_A (133–138) and **b** 3aj4_B (73–78)

In Table S1 of Additional file 1 we list those sequences that appear both in the 12 clusters of Table 1 and in protein structures which are not contained in any of the clusters. We have investigated these structures, and found 454 new clusters. But most of them have very few elements, only two of them have more than 30 elements. With these new clusters the coverage becomes increased to 88 %. In Fig. 19 we show the (*κ*,*τ*) distributions on the stereographically projected two-sphere of the two clusters with more than 30 elements; the initiators are 1ix9_A (133–138) and 3aj4_B (73–78) correspondingly. These two clusters are found by considering the sequences LKGDKL in cluster III and KDCMLQ in cluster XI, respectively.

### Example: Myoglobin

Myoglobin is a widely studied protein, thus we have analysed its loop structure from the present perspective. We have chosen the crystallographic oxymyoglobin structure 1A6M [50] which is one of the few myoglobin structures that have been measured with resolution better than 1.0 Å, for our comparative study.

**Fig. 20:**
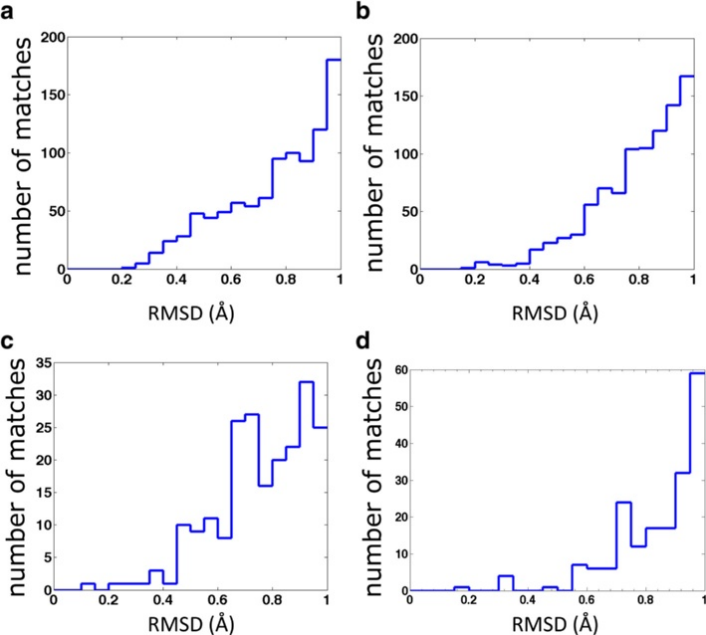
The number of matches for different kinks in myoglobin. In each panel, x-axis is the different RMSD cut-off value (*r*_*rmsd*_) while y-axis is the number of the entries whose RMSD values compared with the initiator are in the range [*r*_*rmsd*_,*r*_*rmsd*_+0.05]. Panels **a**–**d** are for different kinks of myoglobin: **a** (41–46), **b** (48–53), **c** (77–82) and **d** (78–83)

We have located in 1A6M four putative kink segments with six residues each, that are either unique or very rare in PDB, with our 0.2 Å RMSD cut-off. These kinks are located between helices C and D, and between helices E and F. The two putative kinks between helices C and D correspond to the residue sites (41–46) and (48–53). The two putative kinks between helices E and F correspond to residue sites (77–82), and the practically overlapping (78–83). In Fig. 20 we show how in our PDB set, the number of matches for each of these four kinks depends on the RMS cut-off distance.

The 1A6M is closely related to the PDB entries 1A6G, 1A6K and 1A6N; they represent four different ligation states of the same protein. Each of the three 1A6G, 1A6K and 1A6N have been measured with resolution above 1.0 Å, thus they do not appear in our data set. In Table 5 the RMS distance of the four rare kinks of 1A6M are compared to the corresponding kinks in 1A6G, 1A6K and 1A6N. All the RMSD values are below the cut-off 0.2 Å.

**Table 5:** RMS Distance between the four kinks in 1A6M and the corresponding segments in the three other ligation states (in Å ngströms)

Segment	1A6N	1A6K	1A6G
41–46	0.07	0.04	0.17
48–53	0.04	0.02	0.03
77–82	0.04	0.05	0.07
78–83	0.06	0.05	0.07

We conclude that the four kinks are stable, in the sense that they do not change their conformation when the ligation state changes.

### Chain inversion

Finally, the operation of local *chain inversion* along a protein segment is defined as a mapping, that sends a sequence with C *α* coordinates

$$\left\{ \ \mathbf r(i), \ \mathbf r(i+1), \ \ldots \, \ \mathbf r(i+k-1), \ \mathbf r(i+k) \ \right\} $$

into a sequence with C *α* coordinates

$$\{ \ \mathbf r(i+k), \ \mathbf r(i+k-1), \ \ldots \, \ \mathbf r(i+1), \ \mathbf r(i) \ \} $$

We note that a regular secondary structure such as an *α*-helix becomes mapped onto itself *i.e.* remains invariant under chain inversion. But we have found that the 12 clusters that we have constructed are not inversion invariant; the inversion does not map a cluster onto itself. Thus one might expect that new clusters could be found by inversion of these clusters. However, surprisingly we have found only one single example of a PDB segment by inversion. This is the segment (1115–1120) in the PDB structure 1MC2. Thus local chain inversion is apparently a broken symmetry, in the case of protein loops. This sets the loops apart from the regular structures like *α*-helices and *β*-strands.

## Discussion

We have introduced the concept of *loop clustering* to analyse those ultrahigh resolution crystallographic protein structures in PDB, that have been measured with resolution 1.0 Å or less. We have chosen these structures since we expect, that in the case of a ultrahigh resolution measurement there should be less need to introduce structure validation. Thus there should also be less bias towards *a priori* chemical knowledge and stereochemical paradigms, in this subset of PDB proteins. Moreover, our investigation of 2.0 Å subset shows that high resolution is necessary to reveal the clustering structure in the case of protein crystals.

We have inquired to what extent the protein structures can be constructed in a modular fashion. For the modular building blocks we have chosen different parameterisations of the unique kink solution to a generalised discrete nonlinear Schrödinger equation. The precision we have used as a criterion in making a difference between two structures is 0.2 Å in RMSD. We have concluded that this should be the shortest meaningful RMS distance that can be introduced, at the moment, to classify different modular protein components.

We have identified a set of 12 different kink parameterisations, which cover around 38 % of all PDB loop structures. Accordingly, these are loop patterns that are abundantly present in the folded proteins. It appears to us, that these kinks are often located in such protein segments that are structurally important, as opposed to those that are functionally important. We have introduced various techniques to extent the initial set of 12 kinks, and we have found that around 52 % of loop regions become covered when we introduce a set of 29 additional kinks. But in order to cover the remaining ∼48 % of protein loops, we need to substantially increase the number of kinks. For example, we need to introduce over 1000 kinks to cover over 88 % of loops. In particular, we have concluded that there are several kinks that are very rare, even unique, in PDB when we use the present cut-off value. We propose that a rare or even unique kink should have a an important functional rôle, in a protein. This can be exemplified by the myoglobin 1A6M segments (41–46), (48–53) and (78–83) which are all rare. These segments also constitute the CD corner and EF corner in myoglobin, which have been argued to be closely related to the ligand migration process [51, 52].

## Conclusions

Protein loops are built in a modular fashion, in terms of various parametrisations of the kink solution to a generalised version of the discrete nonlinear Schrödinger equation. Most loops can be built from a very small number of modular components, these loops are most likely important for the overall structure of the protein. However, there are also several unique, or very rare loops, which are most likely related to the function. The amino acid sequence does not define the structure uniquely, instead a given sequence can give rise to several different conformations.

## Availability of supporting data

The datasets supporting the result of this article are available in Protein Data Bank (PDB) by confining the resolution better than 1.0 Å (http://www.rcsb.org).

## Additional file

Additional file 1
**Description on Supplemental Material.** **Figure S1.** The stereographic distribution map of C _*α*_ atoms in the PDB subset with resolution better than 1.0 Å, which is the same as that of resolution better than 2.0 Å (See Fig. 4). **Figure S2.** and **Figure S3.** The distributions of the amino acids on each site of the six-site-long segments of the clusters listed in Tables 1 and 3. **Table S1.** Sequences that appear both in the 12 clusters and in protein structures which are not contained in the clusters before percolation. (PDF 1178 kb)

## Abbreviations

DNLS: Discrete Nonlinear Schrö
dinger; PDB: Protein Data Bank
RMS: Root-mean-square
CASP: Critical Assessment for Structural Prediction
