# Supplementary material for: Clustering and percolation in protein loop structures
Source: BMC Struct Biol. 2015 Oct 29;15:22. doi: 10.1186/s12900-015-0049-x (PMC4625449; doi:10.1186/s12900-015-0049-x)
Supplement: Additional file 1 — Description on Supplemental Material. Figure S1. The stereographic distribution map of C α atoms in the PDB subset with resolution better than 1.0 Å, which is the same as that of resolution better than 2.0 Å (See Fig. 4). Figure S2. and Figure S3. The distributions of the amino acids on each site of the six-site-long segments of the clusters listed in Tables 1 and 3. Table S1. Sequences that appear both in the 12 clusters and in protein structures which are not contained in the clusters before percolation. (PDF 1178 kb) [file 12900_2015_49_MOESM1_ESM.pdf]

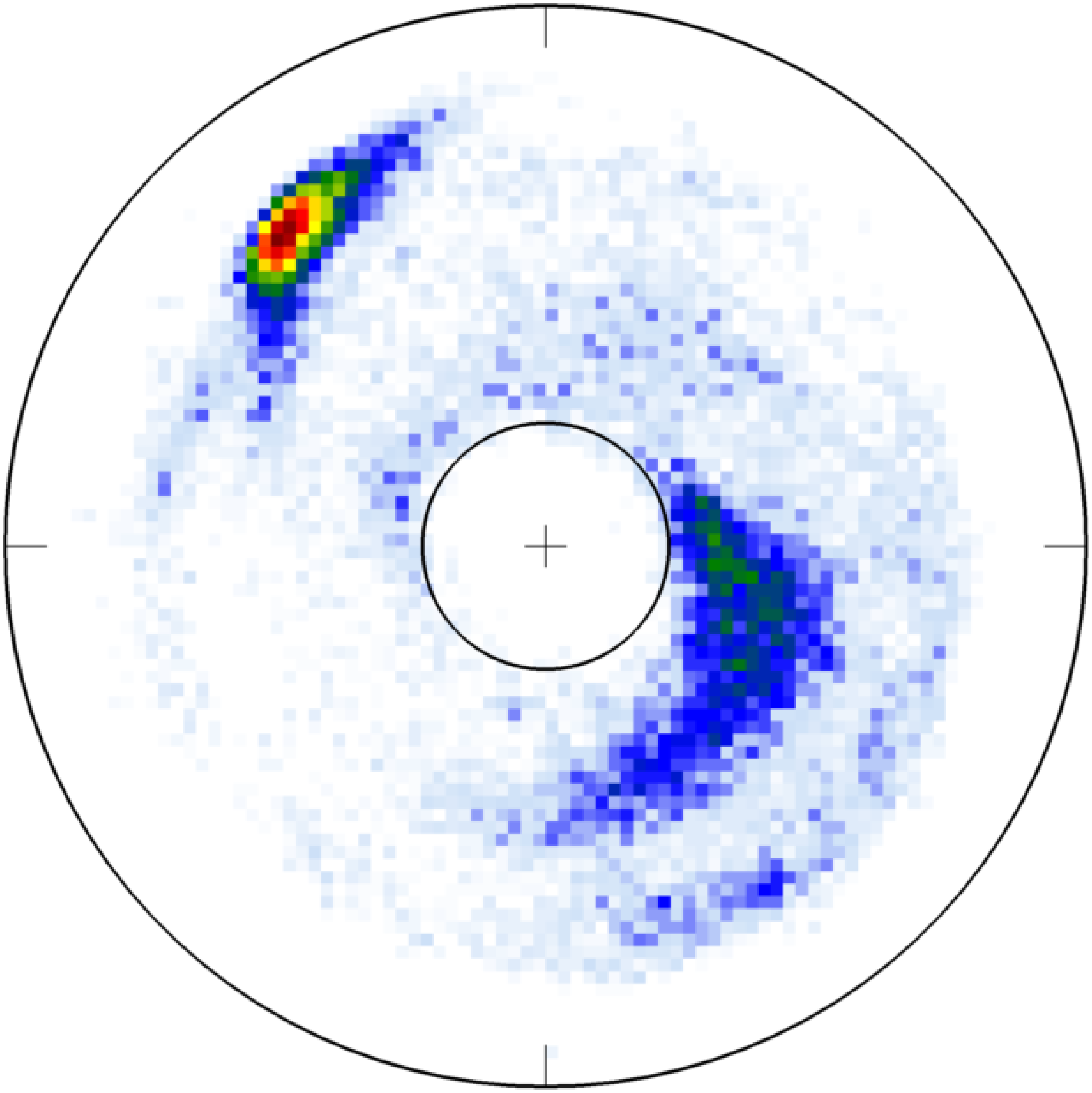

Figure S1: The C_α atom stereographic projection map with PDB subset with resolution better than 1.0 Å . We can see that the pattern of the distribution is the same as Figure 4a) in the main text. Note that the distribution is not so clear as Figure 4a) because the dataset with resolution better than 1.0 Å contains fewer entries.

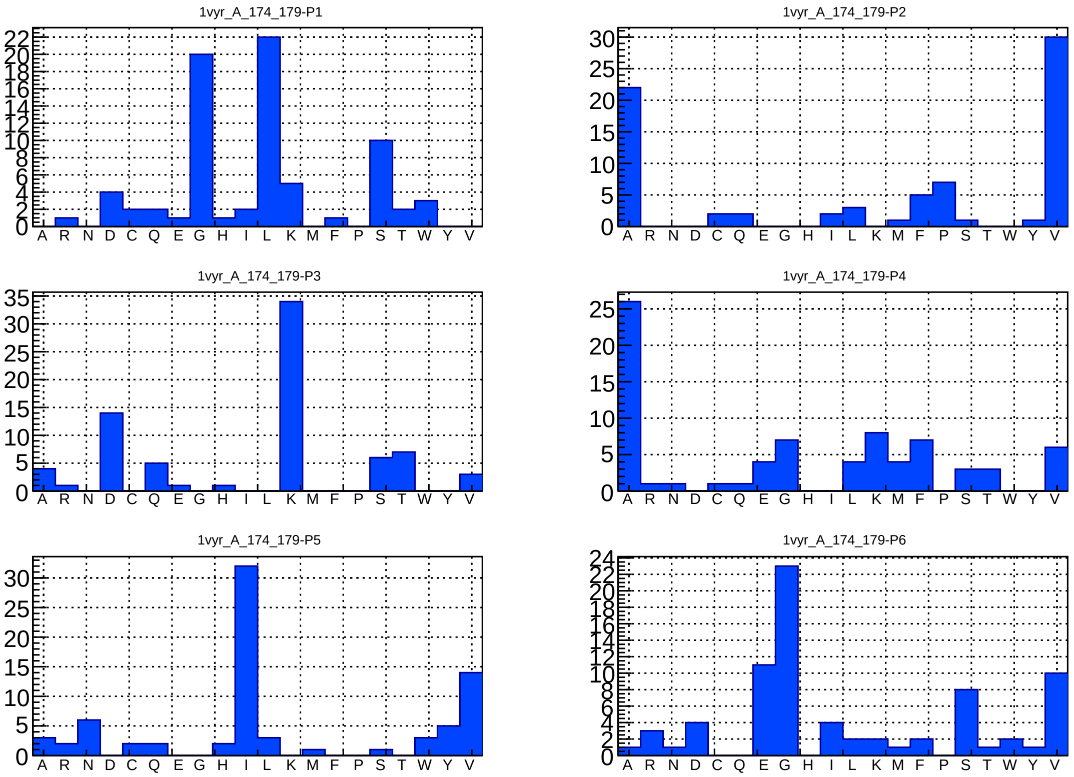

(i) cluster I

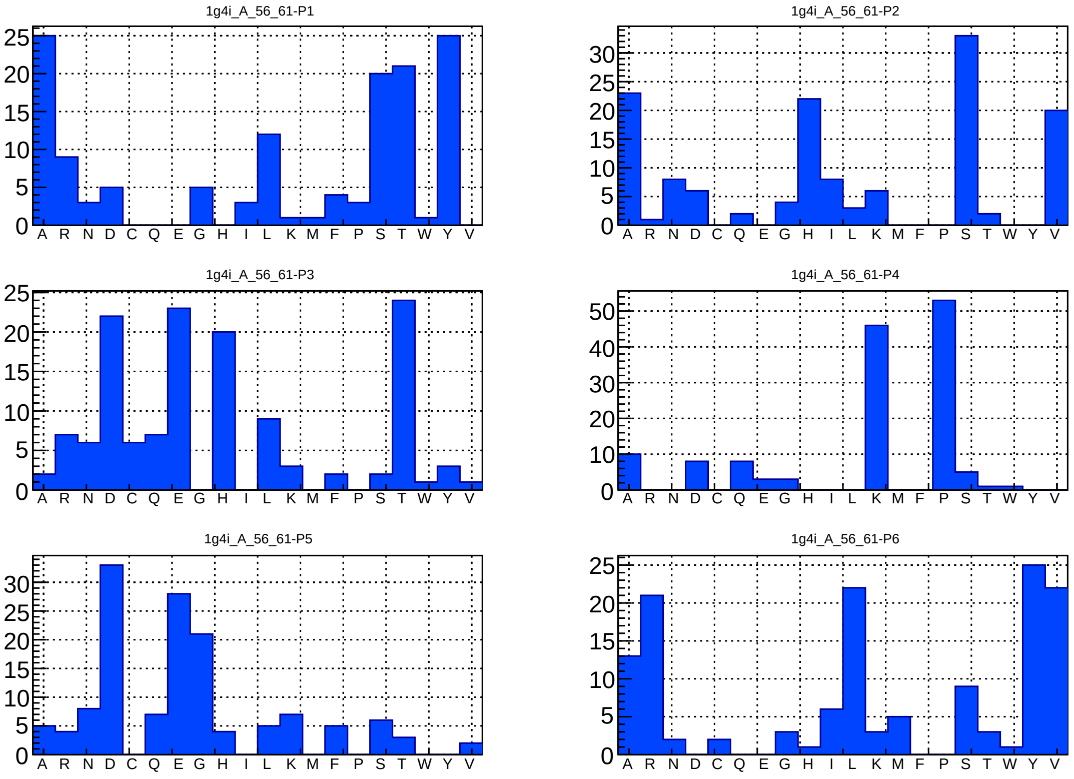

(ii) cluster II

Figure S2

Continued

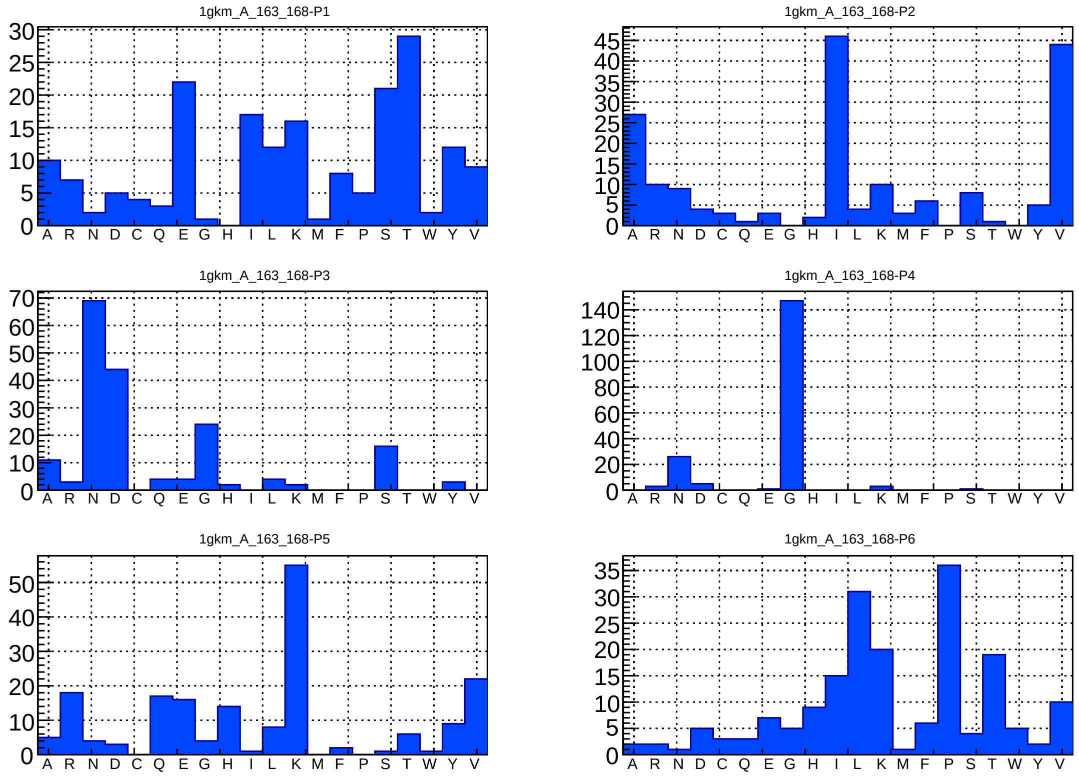

(iii) cluster III

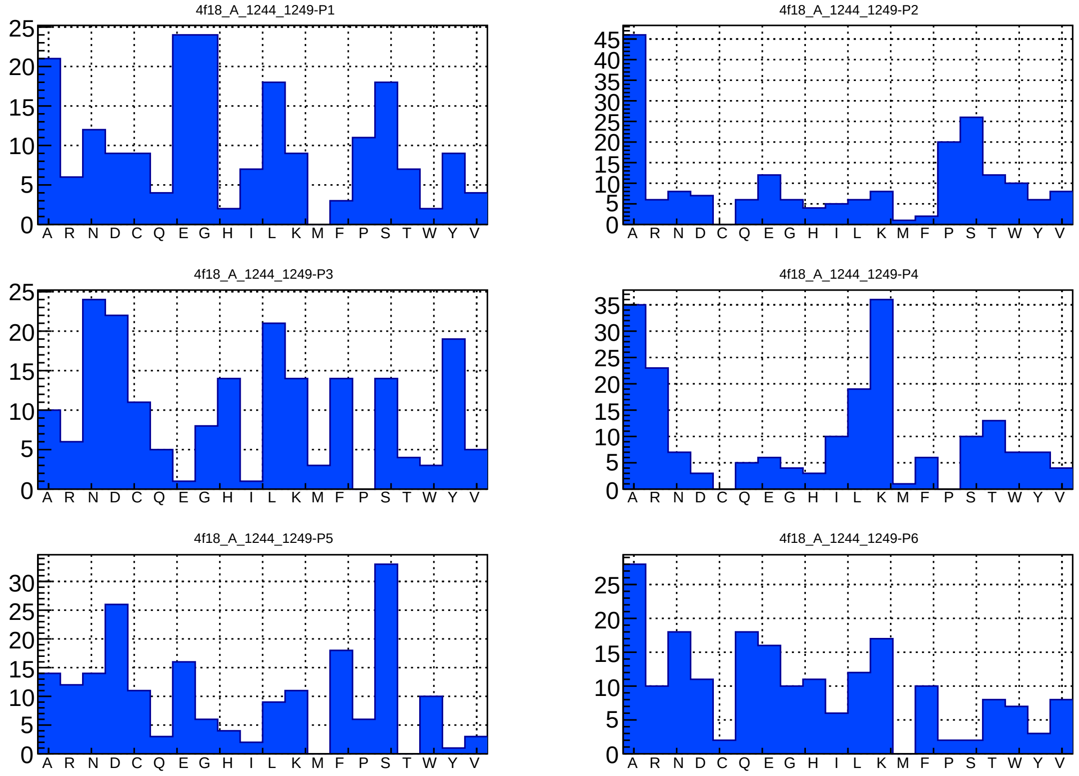

(iv) cluster IV

Figure S2

Continued

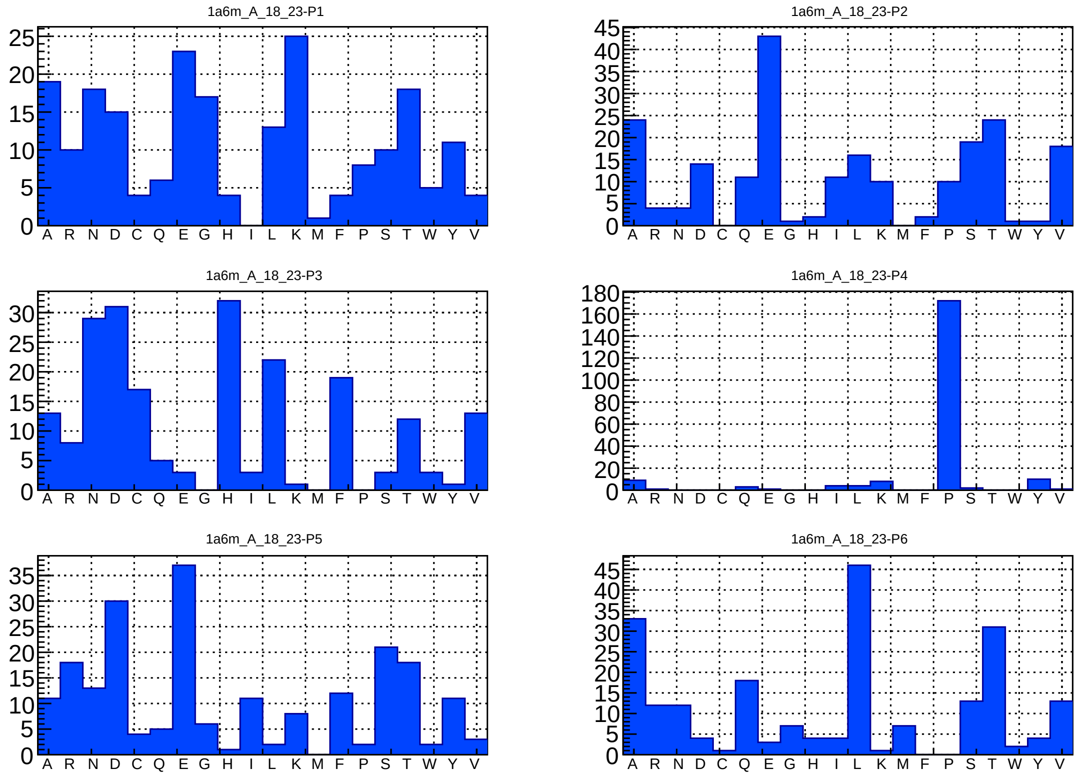

(v) cluster V

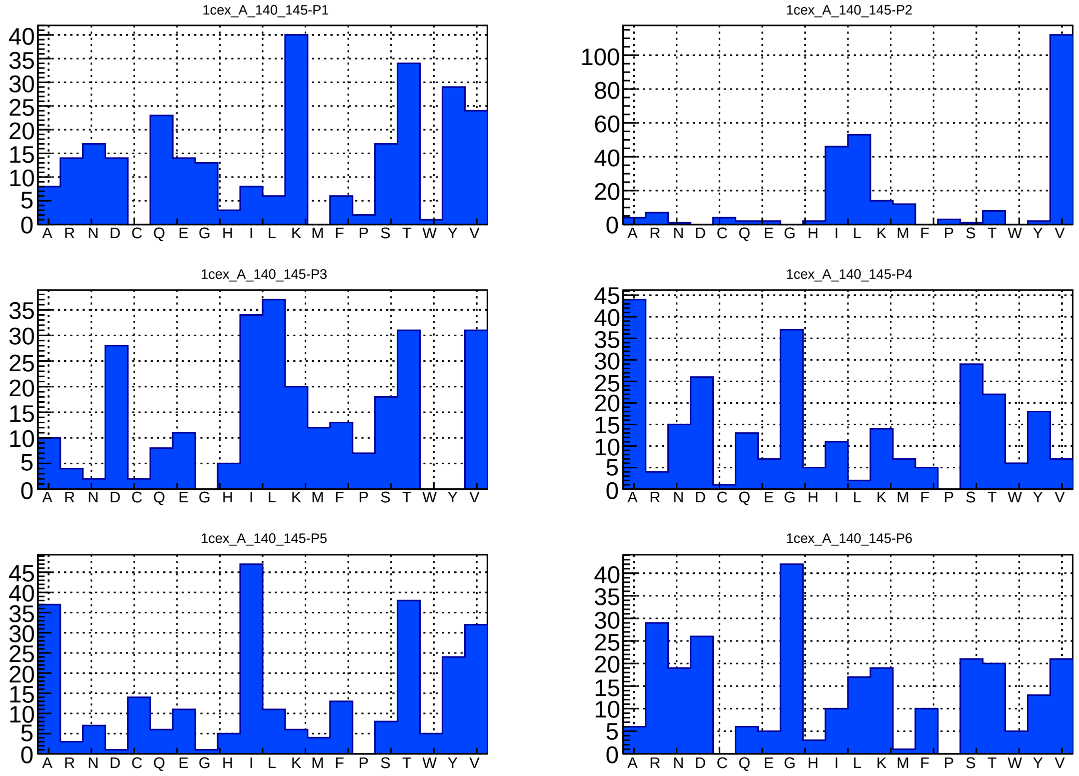

(vi) cluster VI

Figure S2

Continued

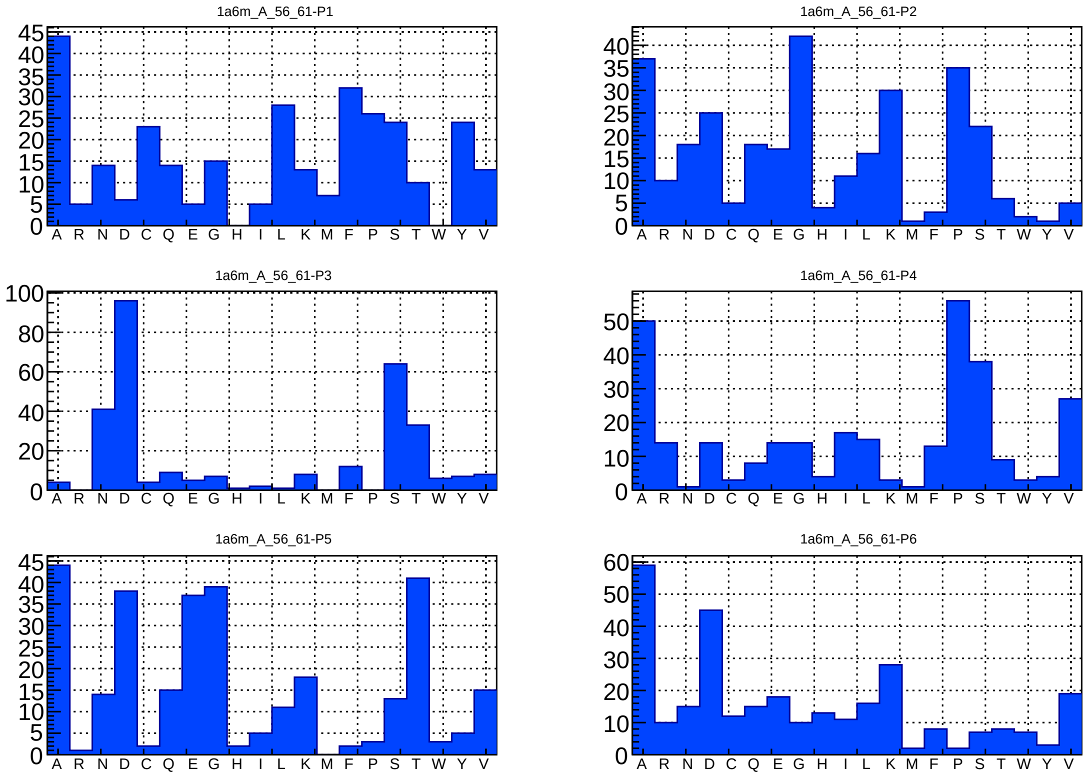

(vii) cluster VII

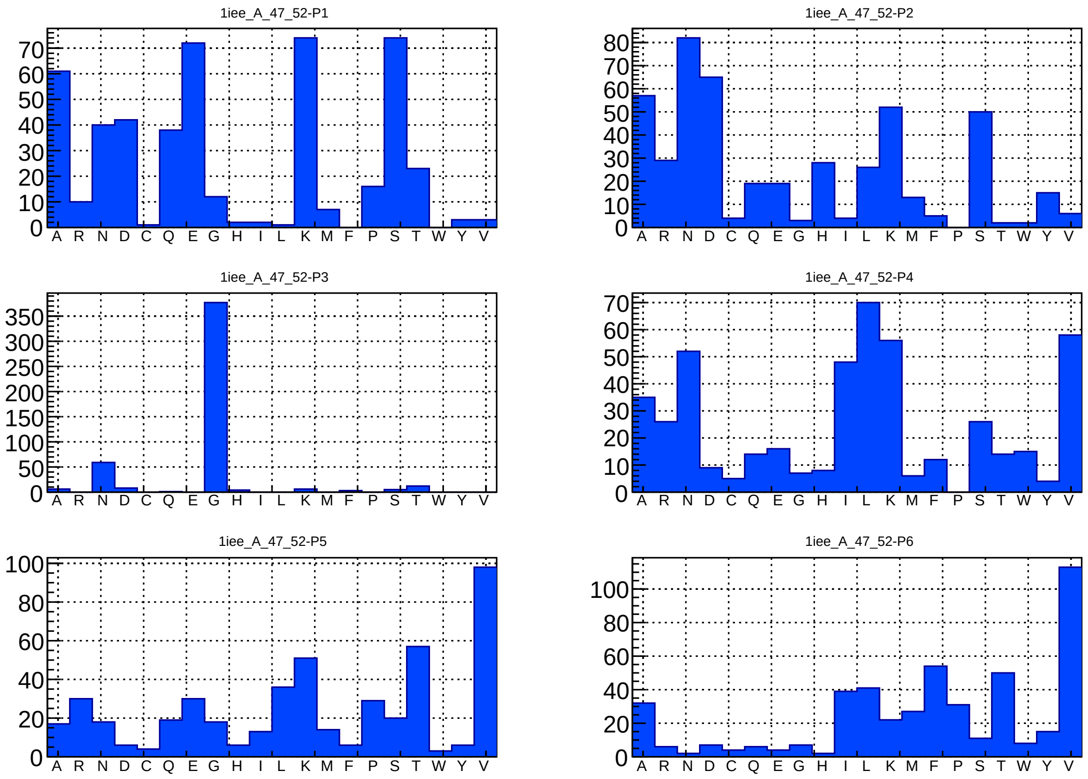

(viii) cluster VIII

Figure S2

Continued

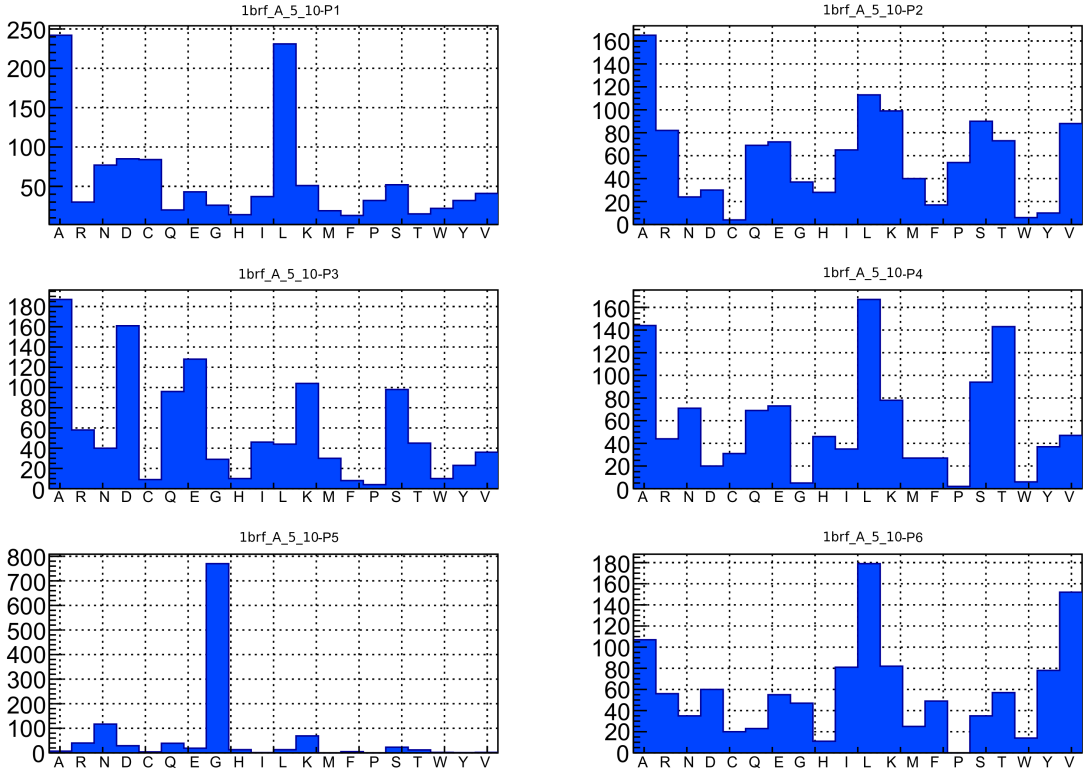

(ix) cluster IX

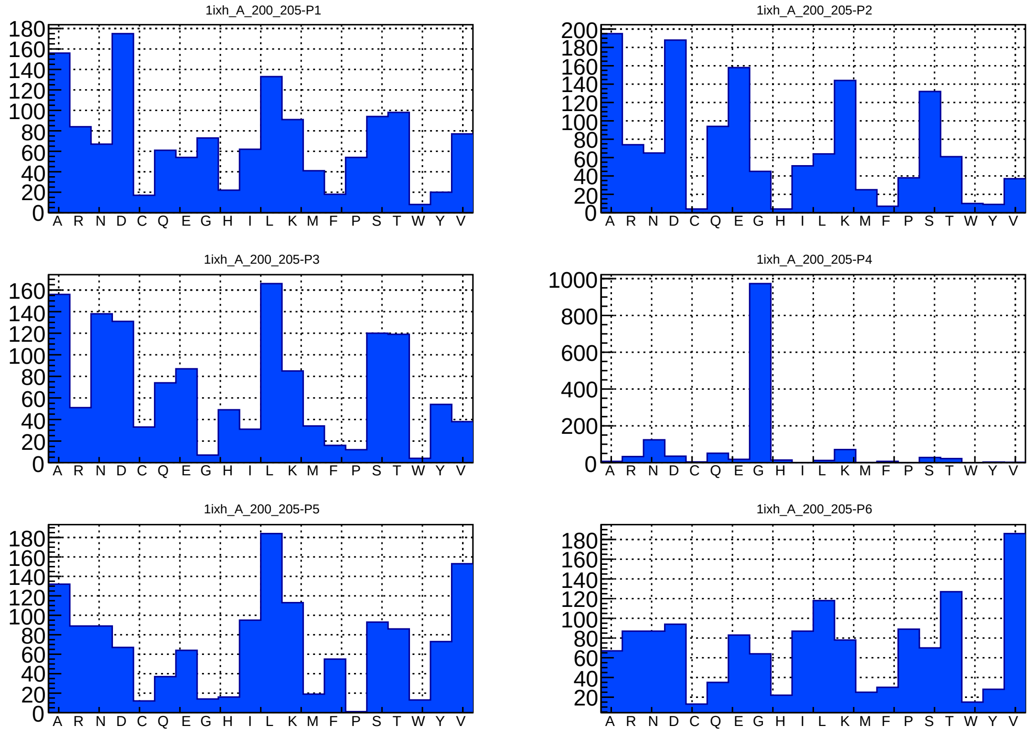

(x) cluster X

Figure S2

Continued

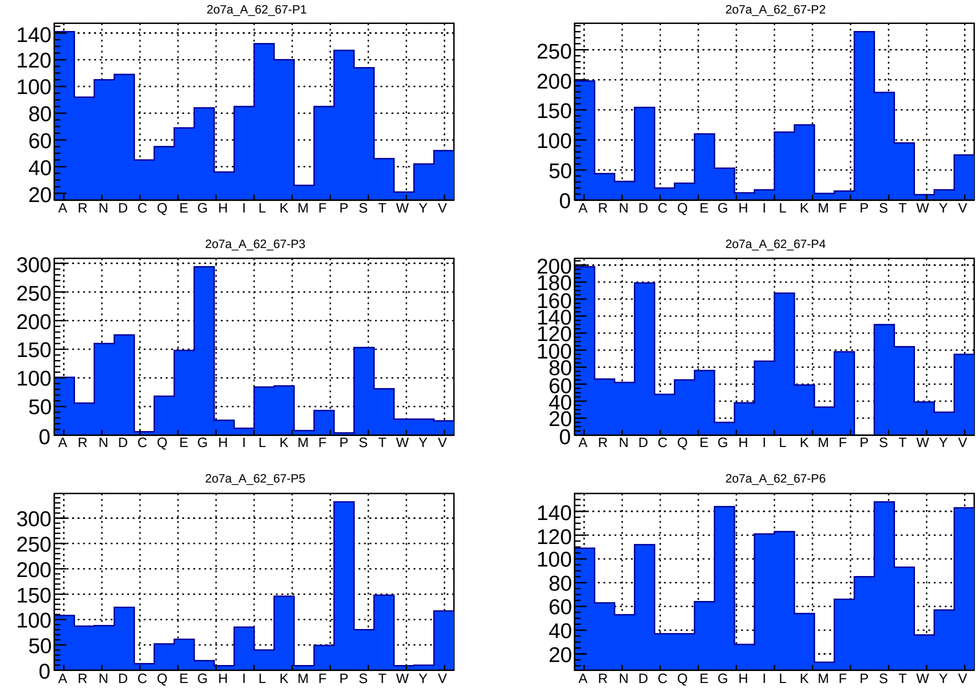

(xi) cluster XI

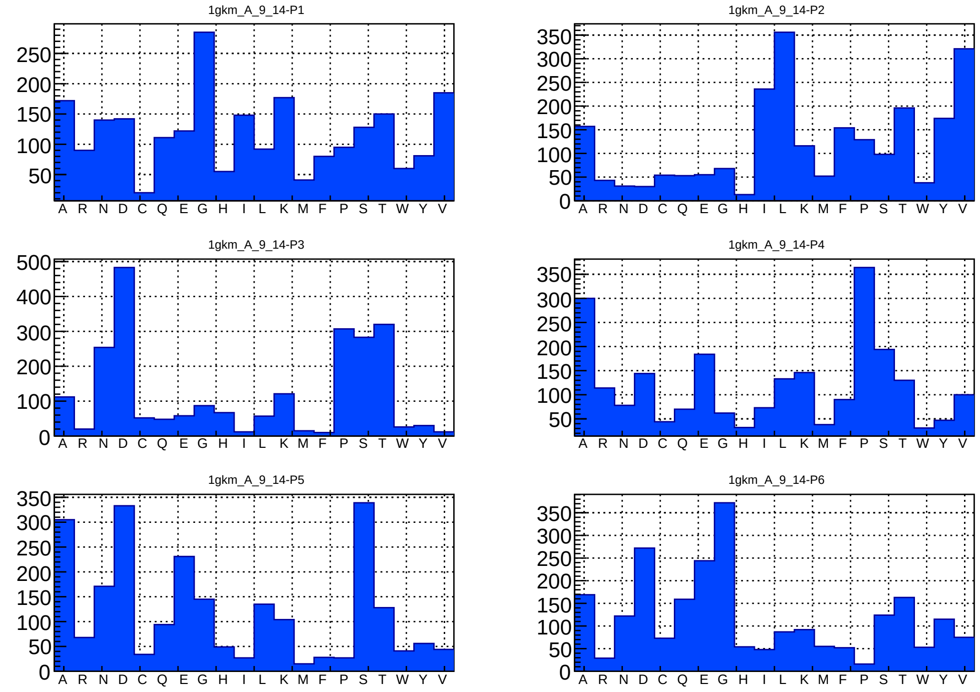

(xii) cluster XII

Figure S2: Amino acids distributions for 12 big clusters. The subfigures are for different clusters. The panels of each subfigure are the distributions of the amino acid for different sites (P1 - P6 represent site 1 - 6 of the segment), where the amino acid are abbreviated in one-letter form. From the amino acid distributions we can see that there are clear patterns in the distributions of the amino acids, especailly for the middle site (P3 and P4) of the segment in each cluster.

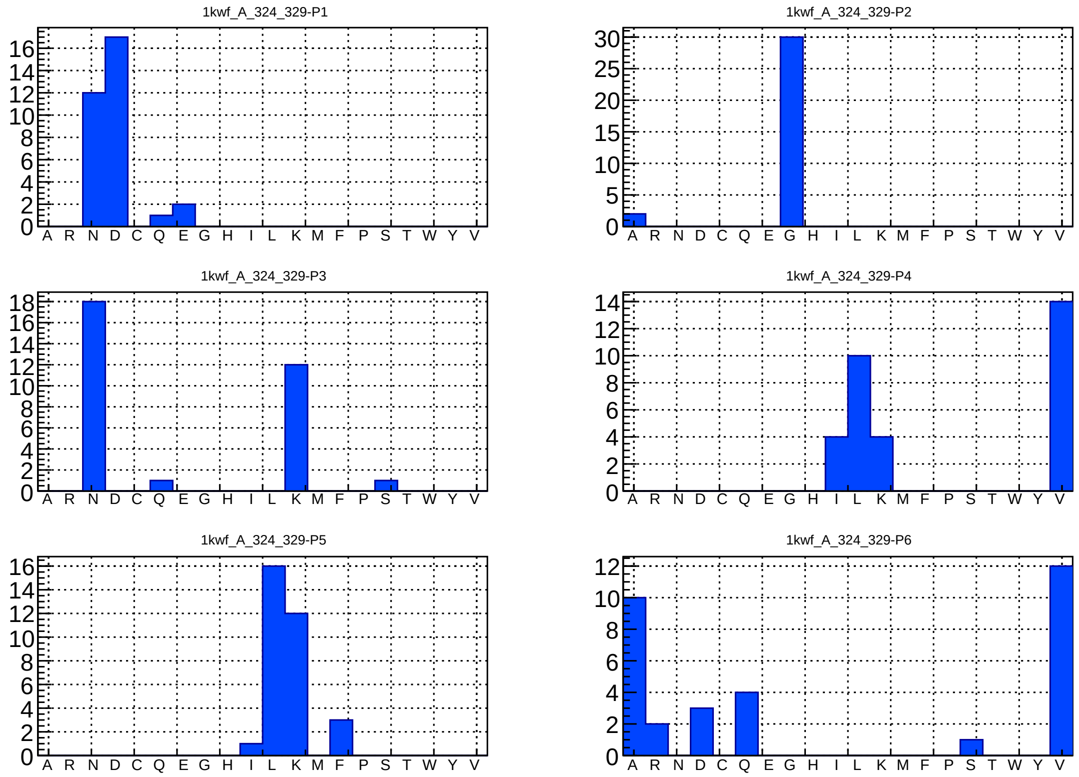

(i) cluster 1

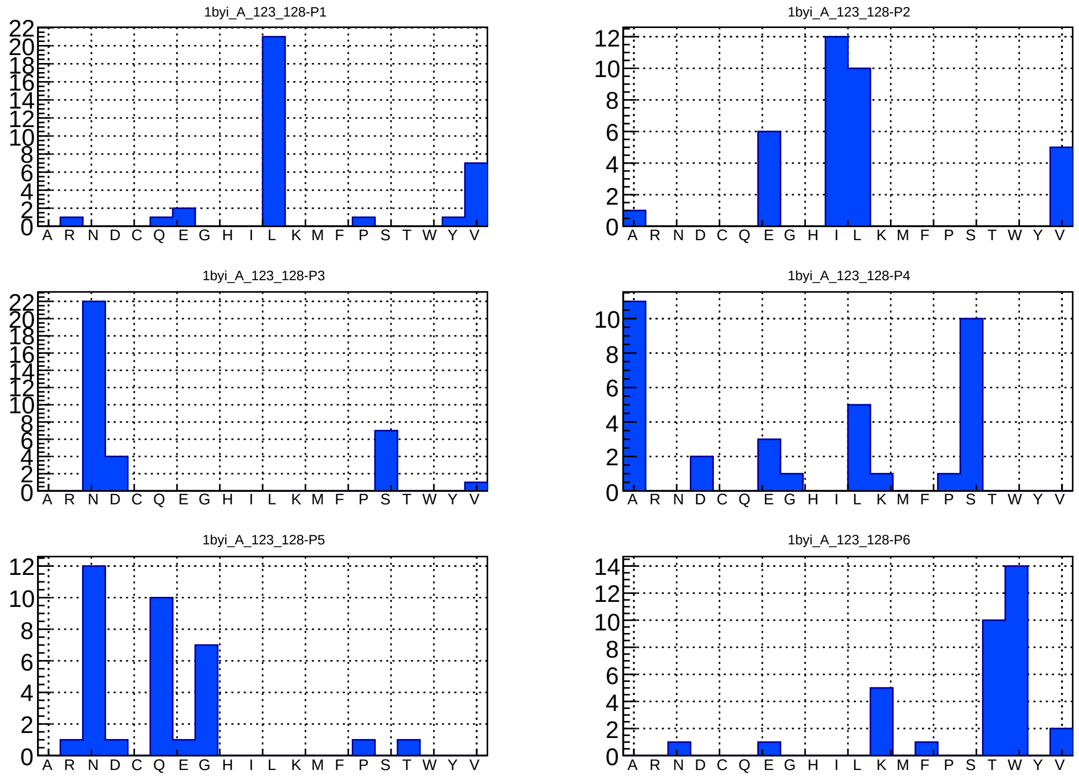

(ii) cluster 2

Figure S3

Continued

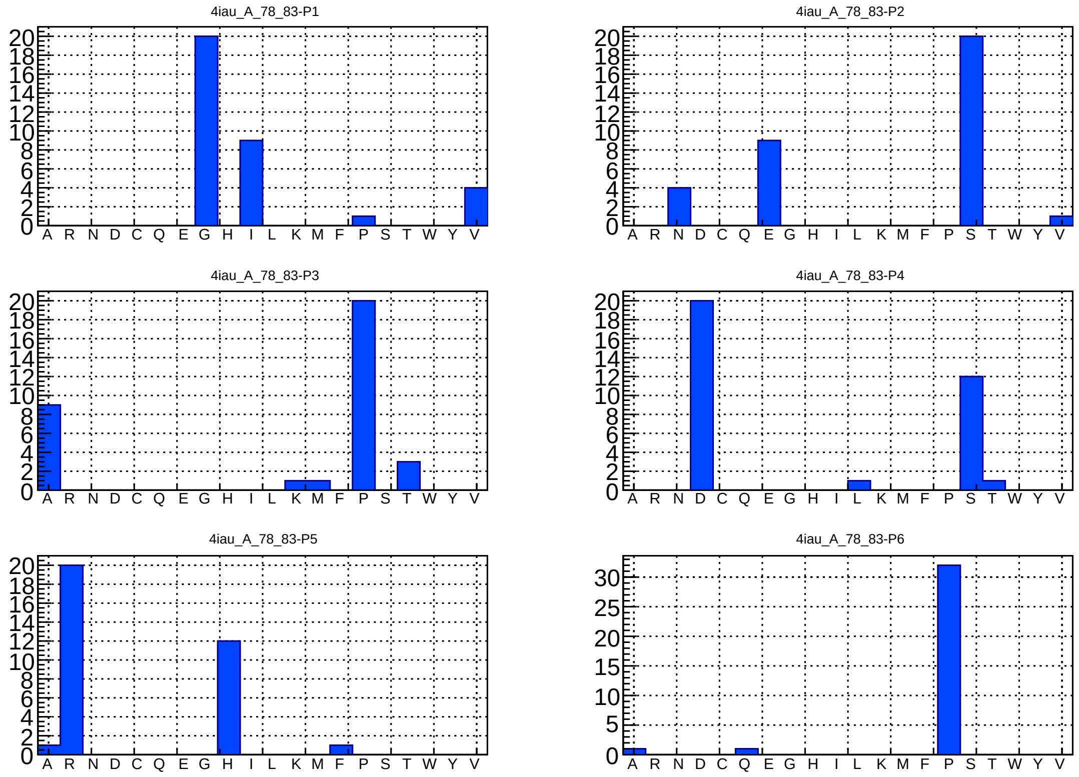

(iii) cluster 3

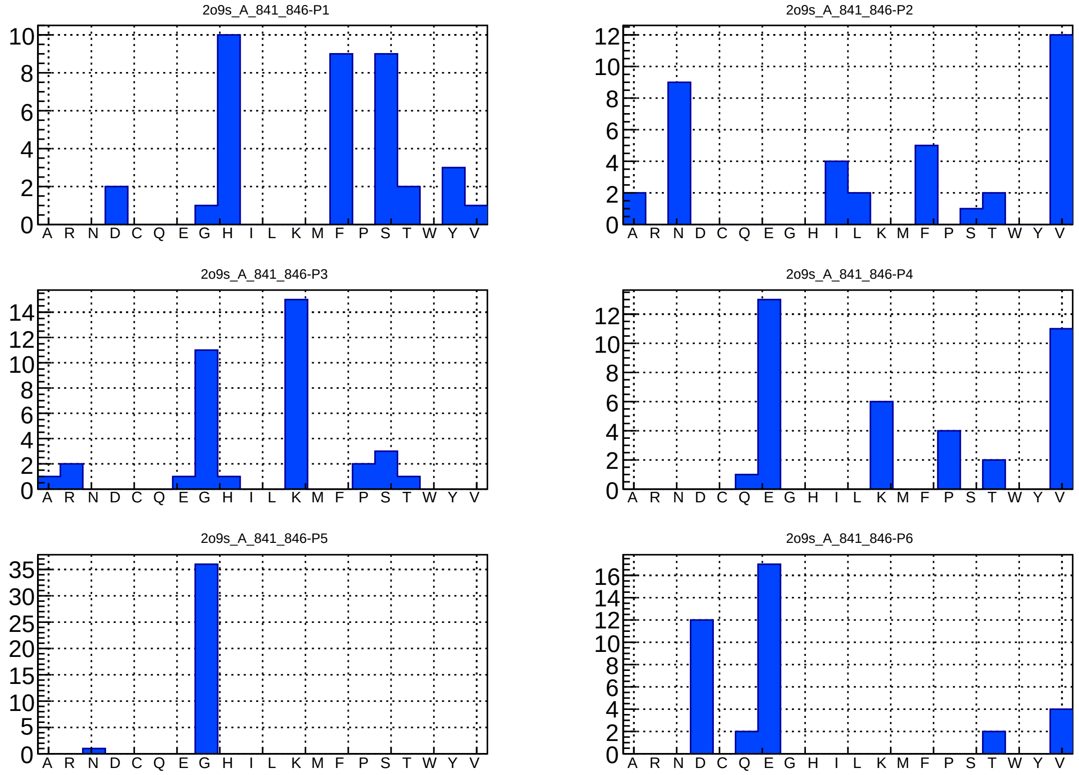

(iv) cluster 4

Figure S3

Continued

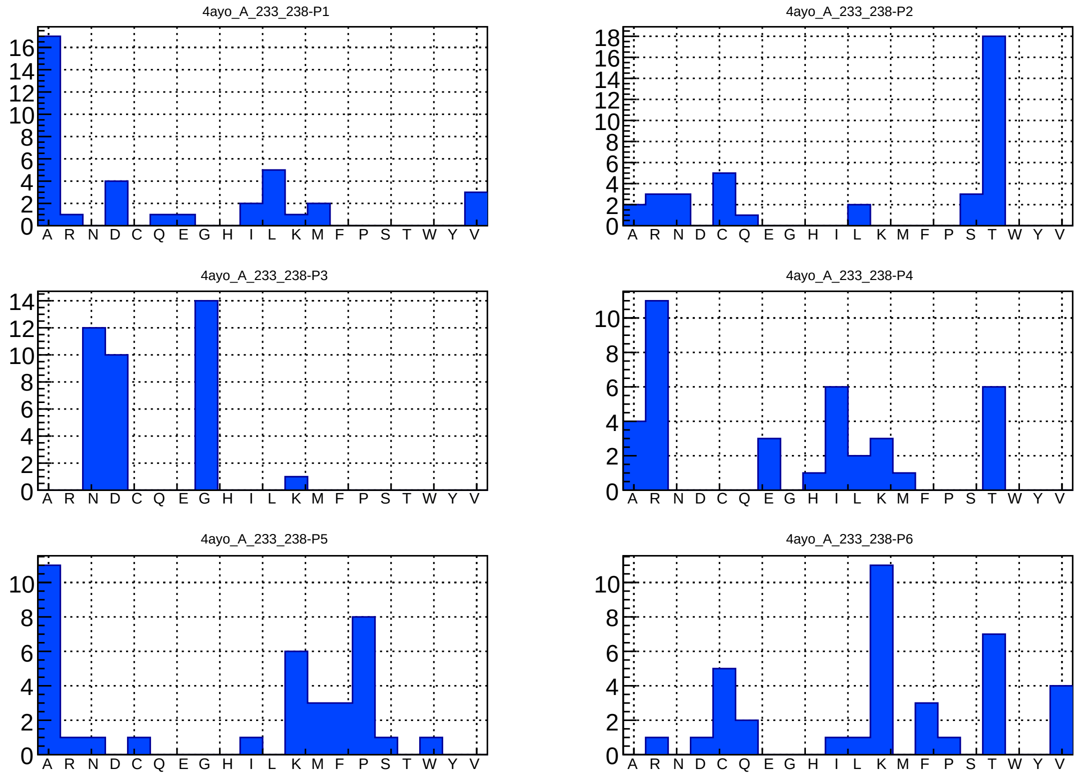

(v) cluster 5

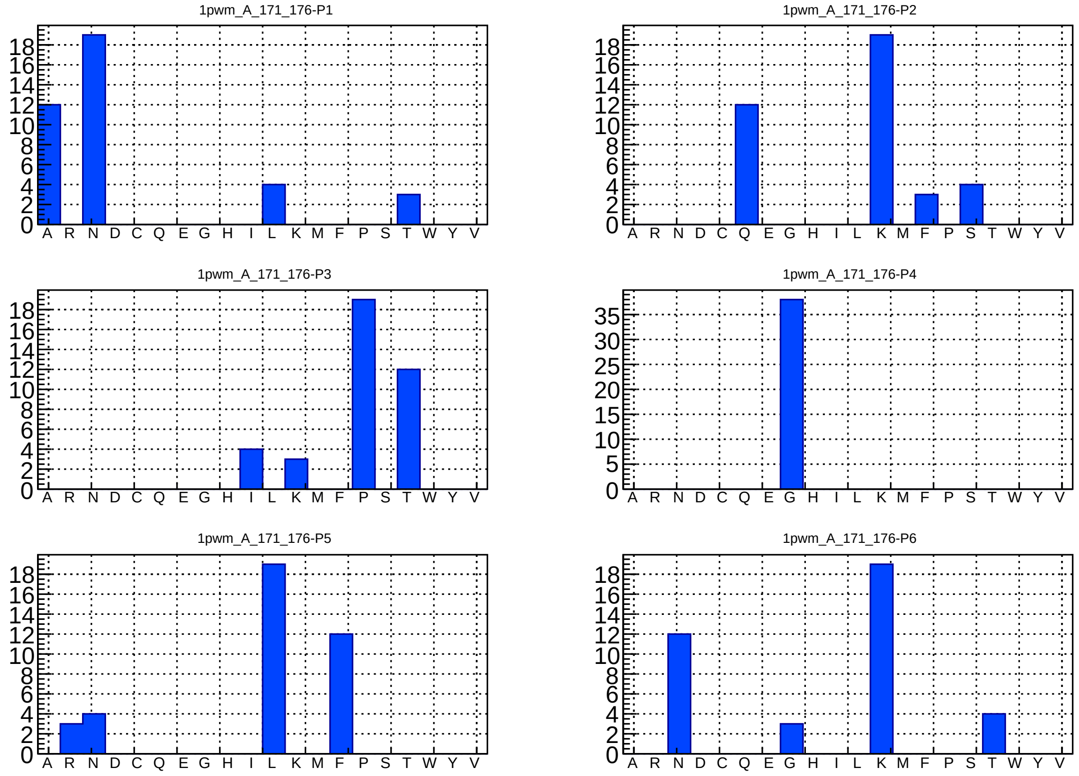

(vi) cluster 6

Figure S3

Continued

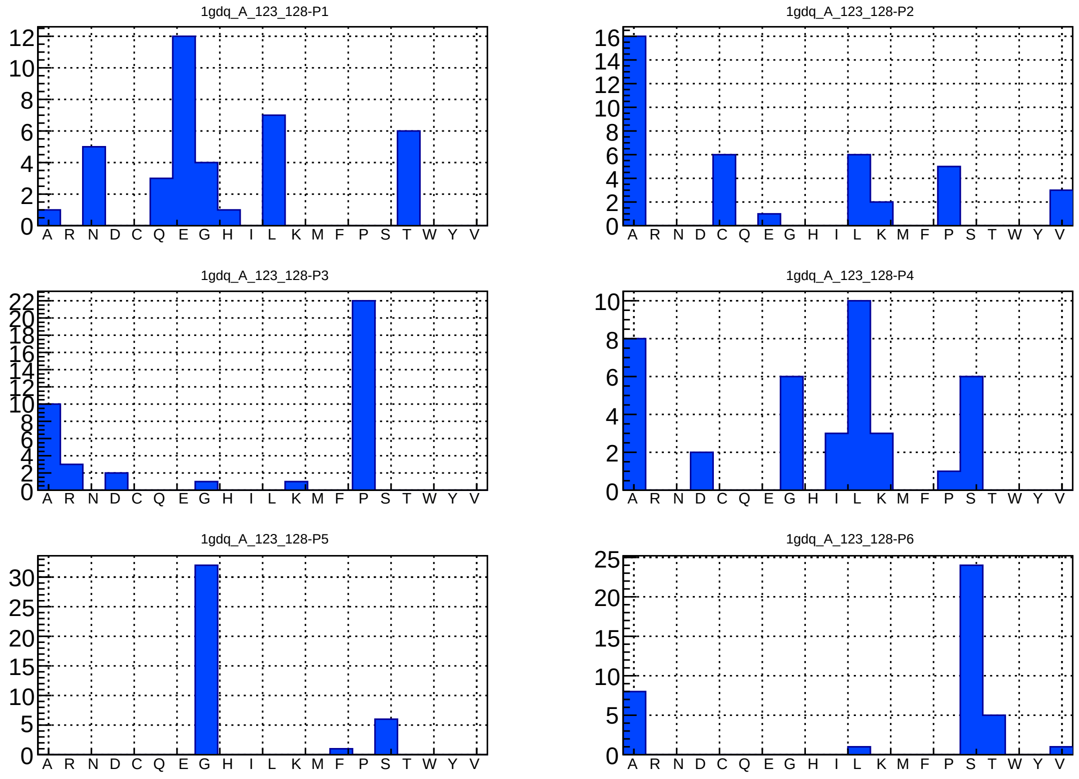

(vii) cluster 7

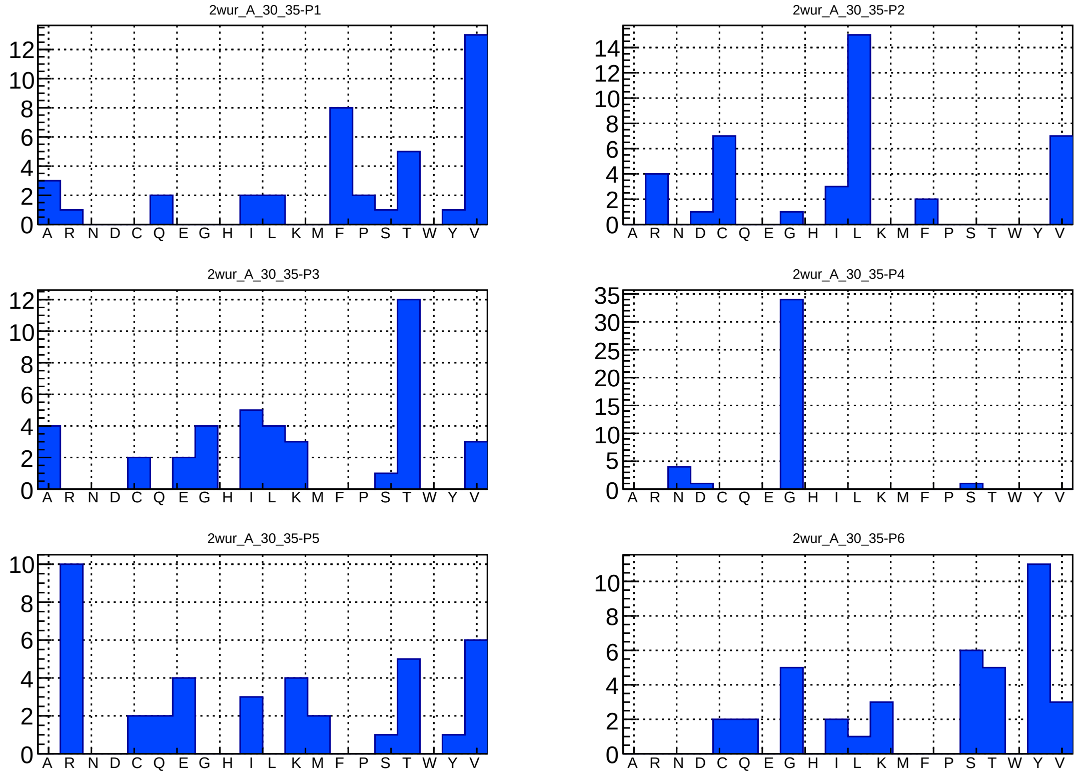

(viii) cluster 8

Figure S3

Continued

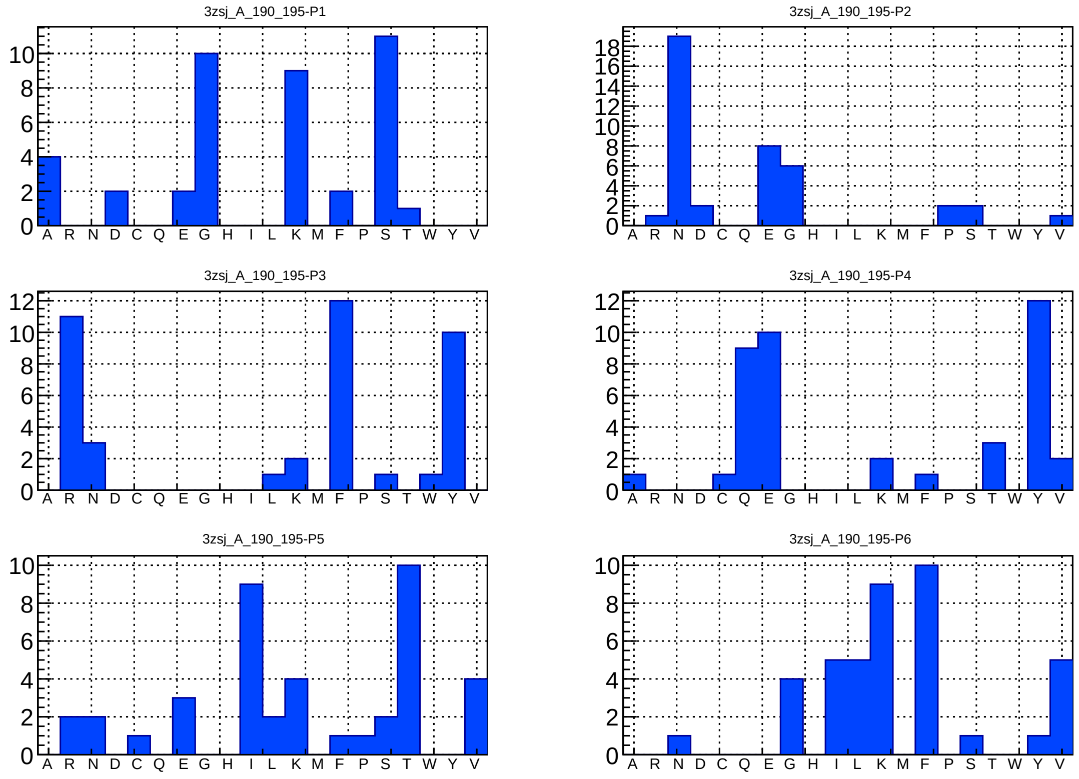

(ix) cluster 9

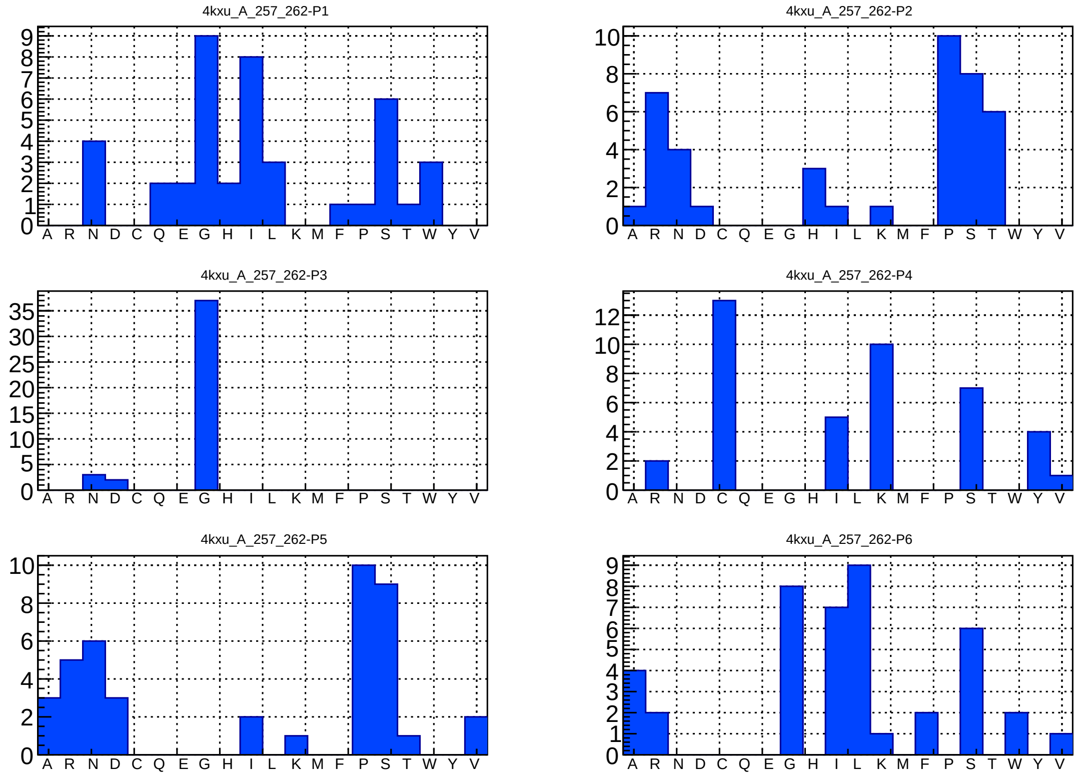

(x) cluster 10

Figure S3

Continued

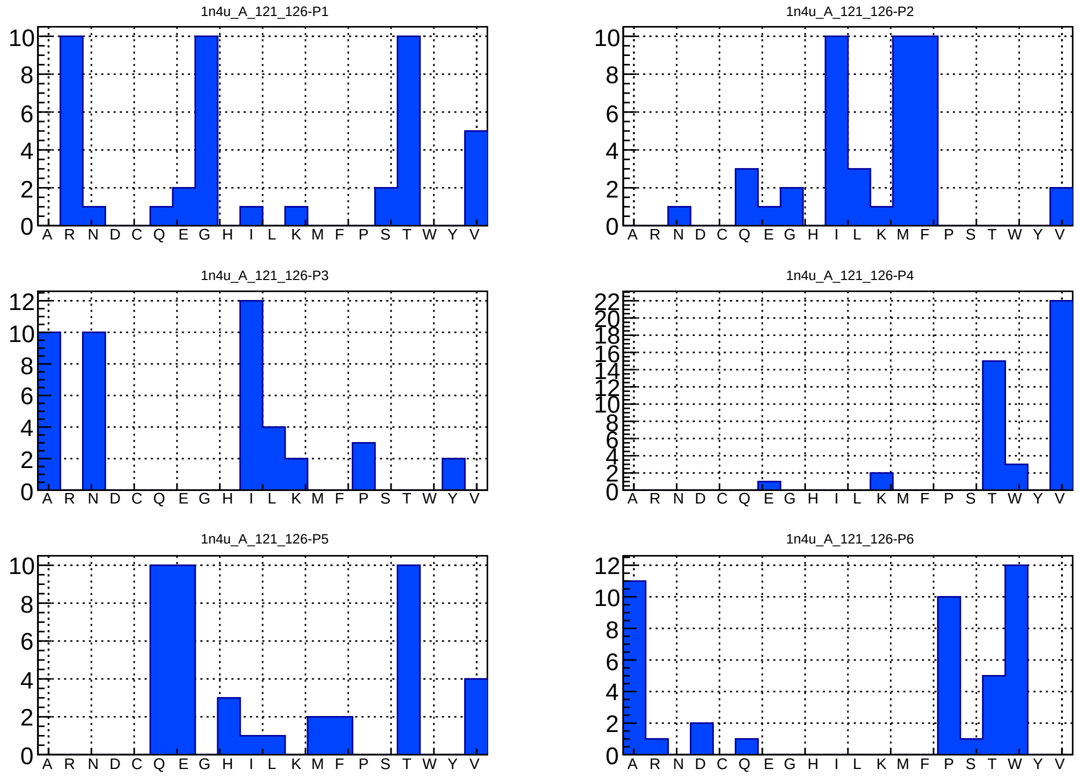

(xi) cluster 11

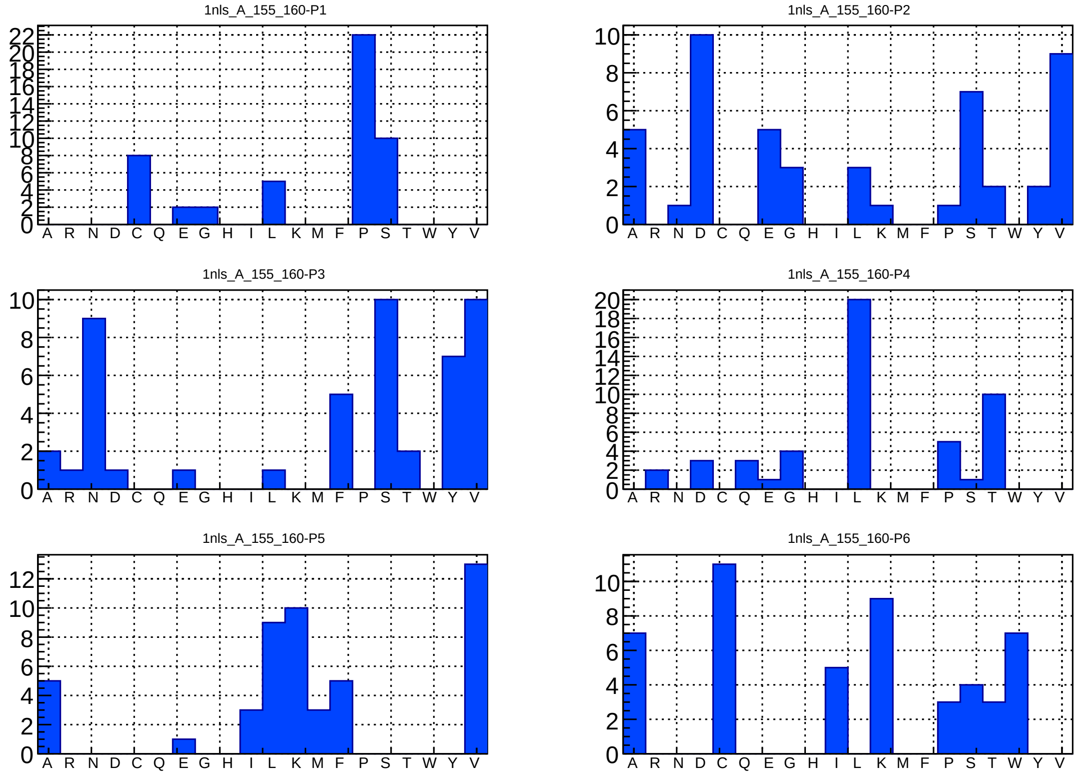

(xii) cluster 12

Figure S3

Continued

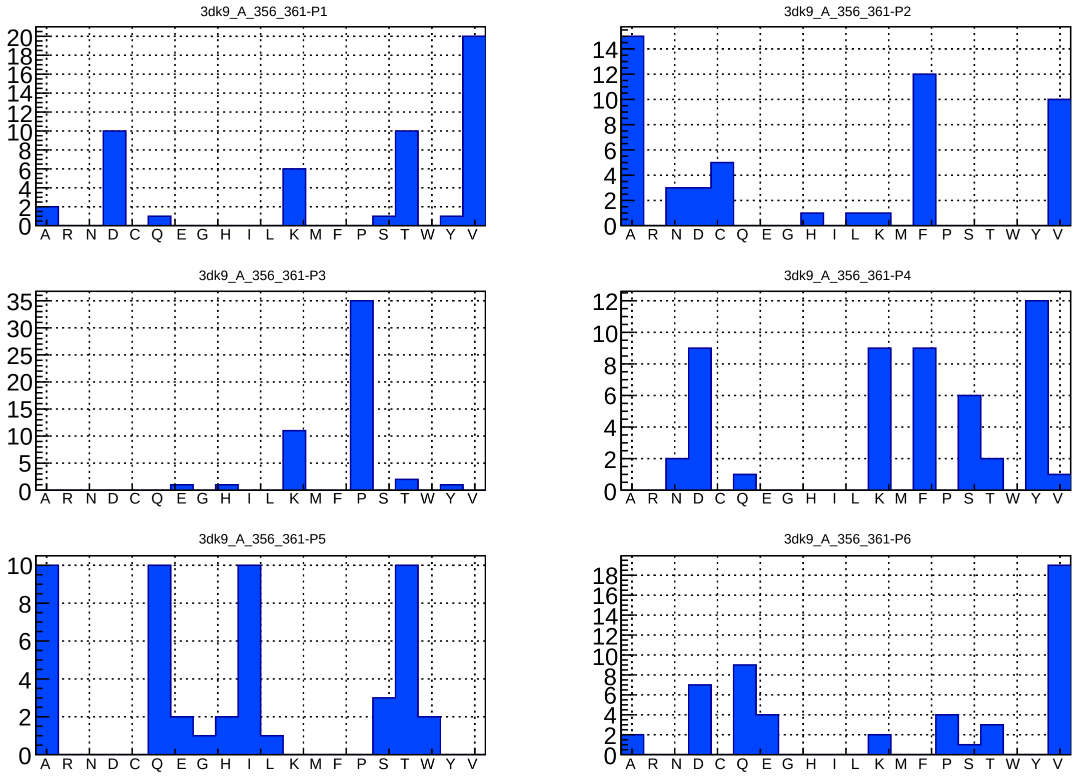

(xiii) cluster 13

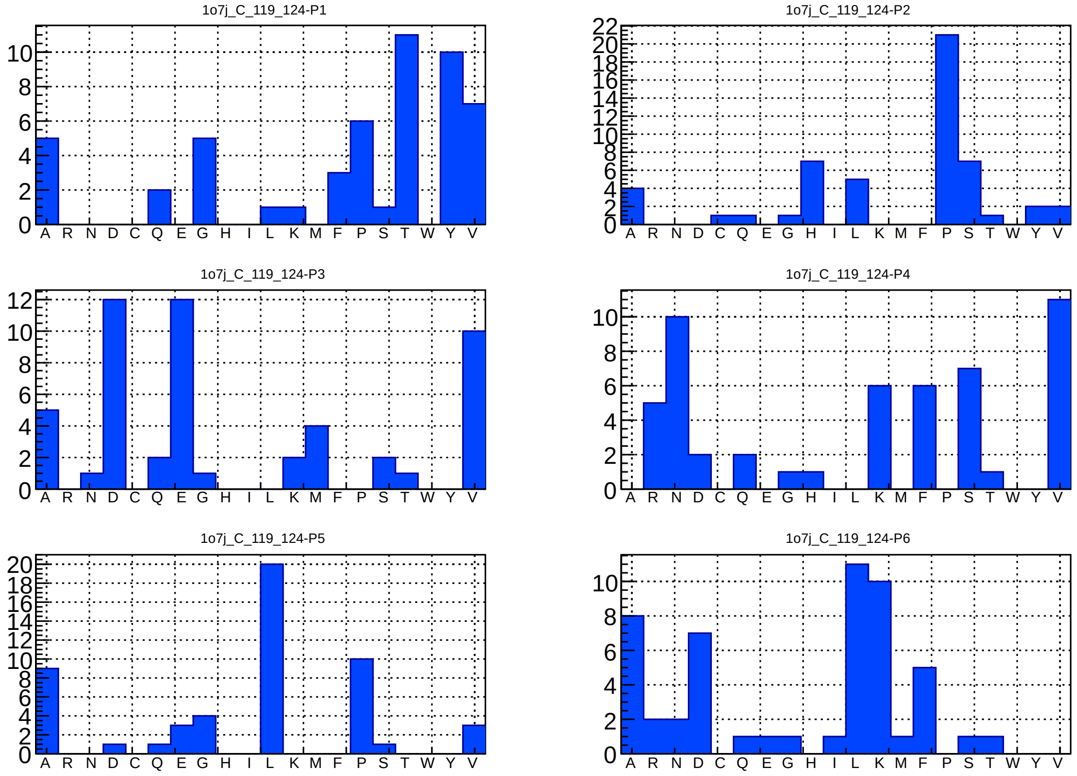

(xiv) cluster 14

Figure S3

Continued

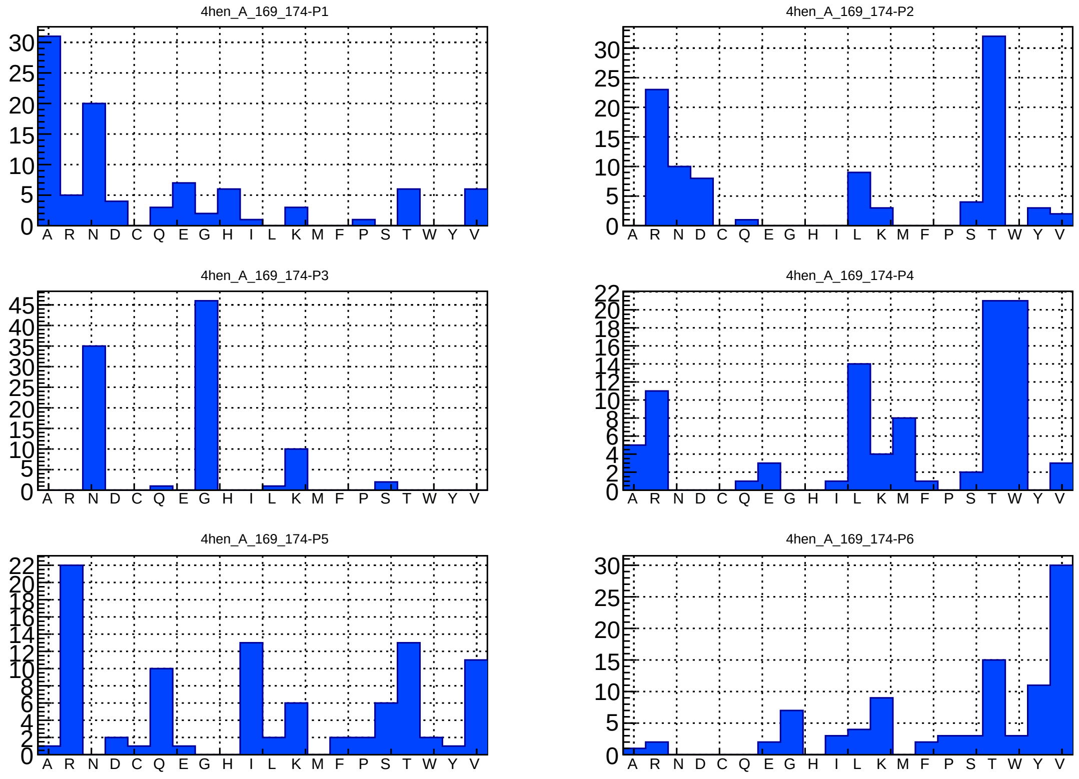

(xv) cluster 15

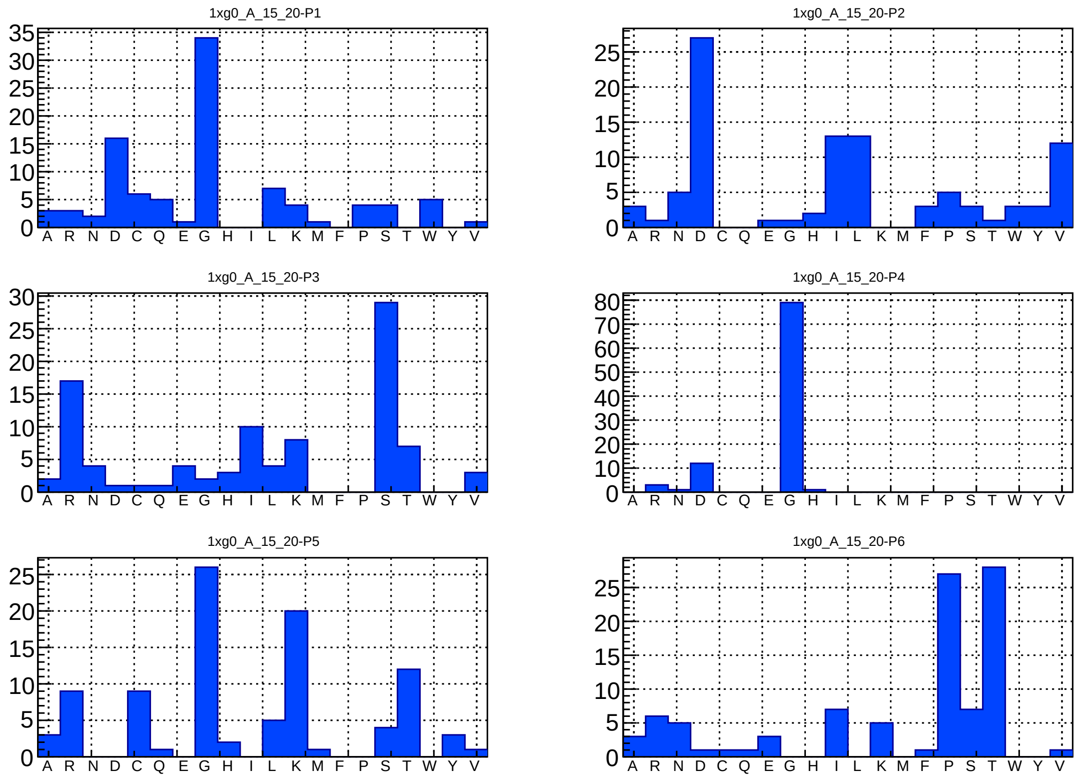

(xvi) cluster 16

Figure S3

Continued

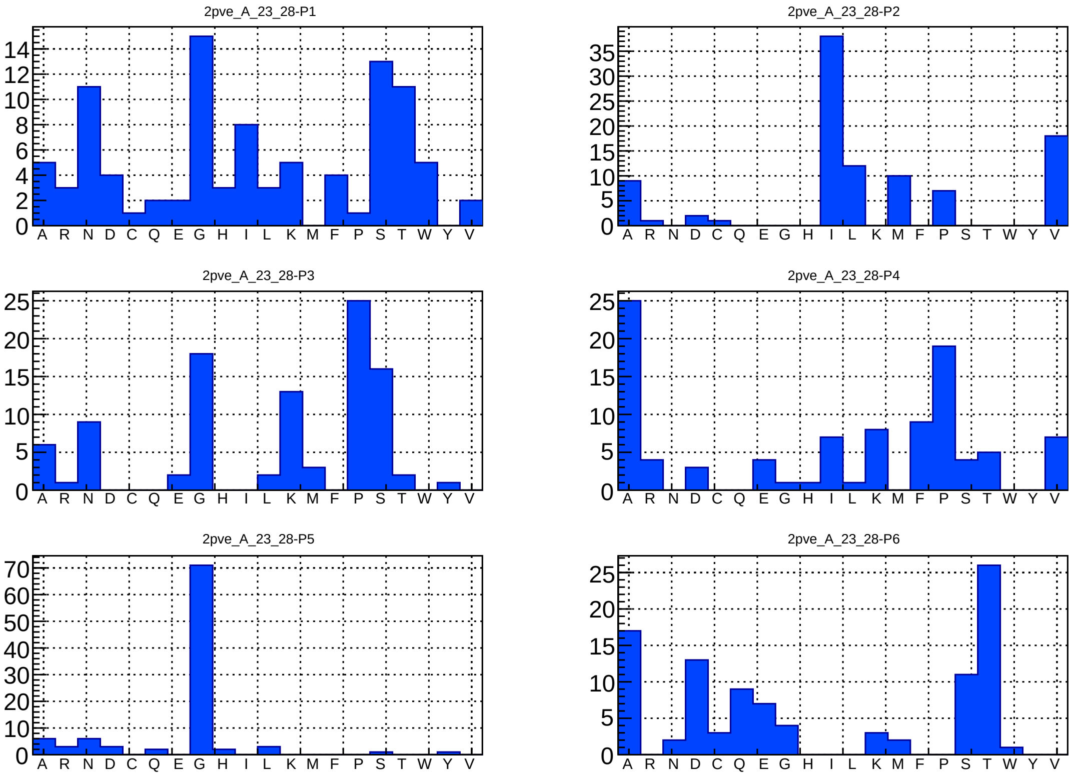

(xvii) cluster 17

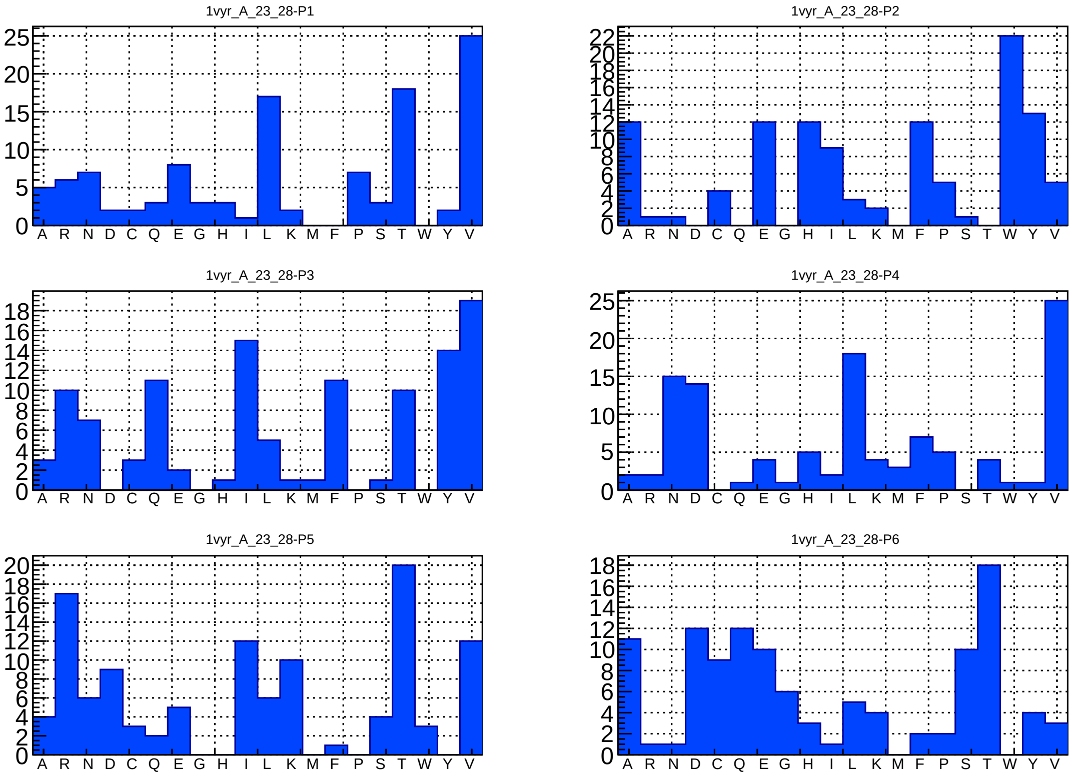

(xviii) cluster 18

Figure S3

Continued

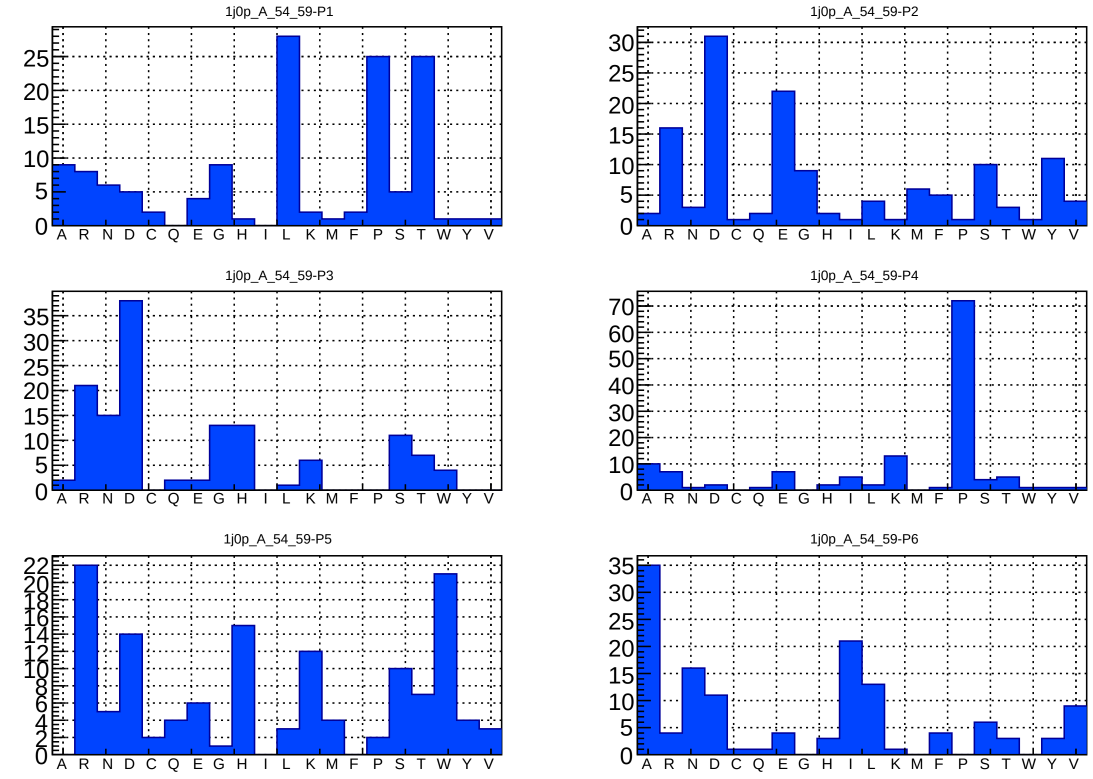

(xix) cluster 19

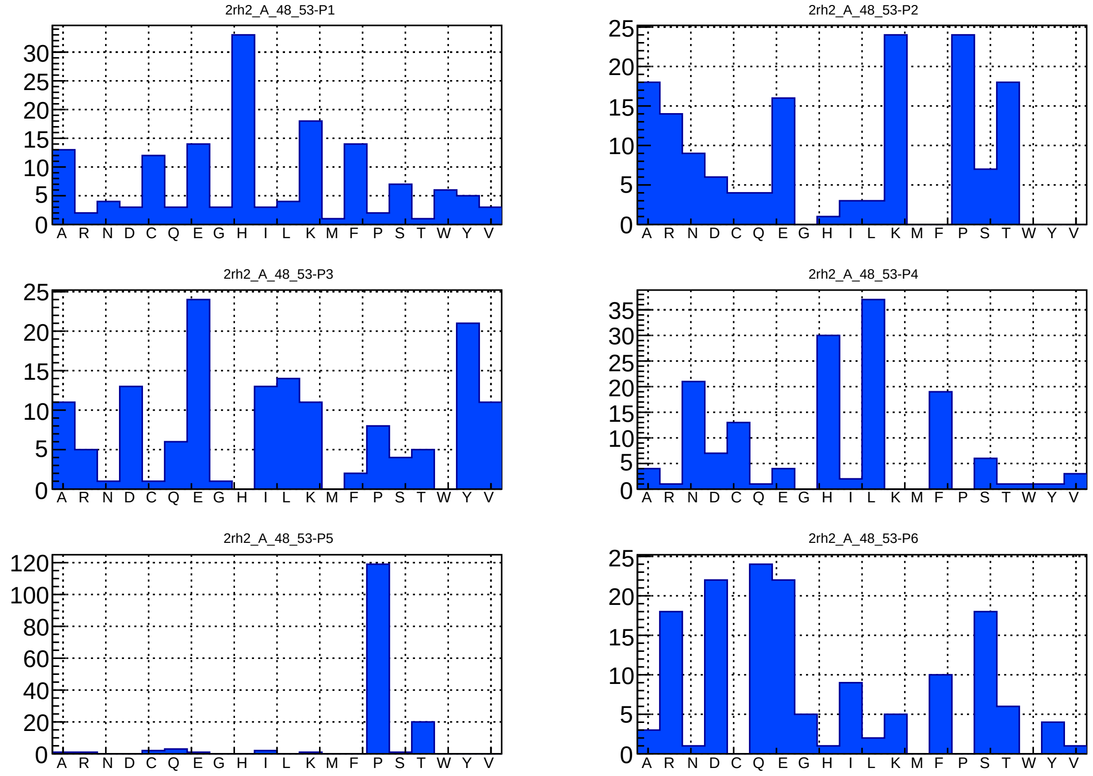

(xx) cluster 20

Figure S3

Continued

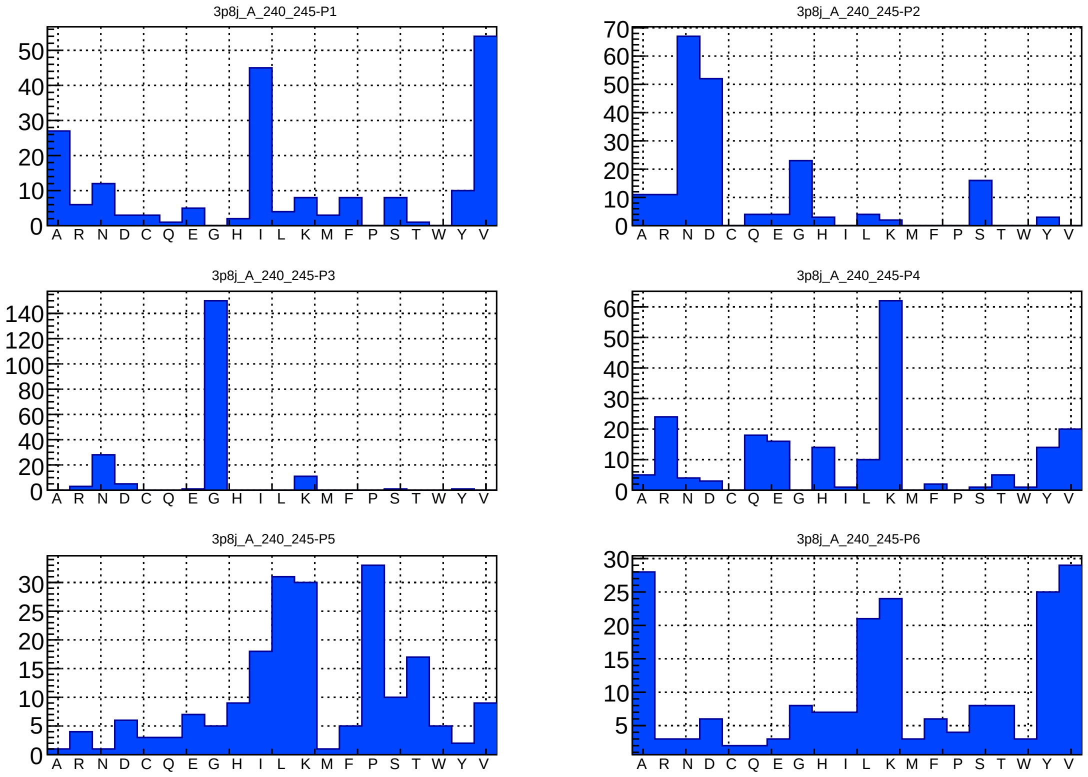

(xxi) cluster 21

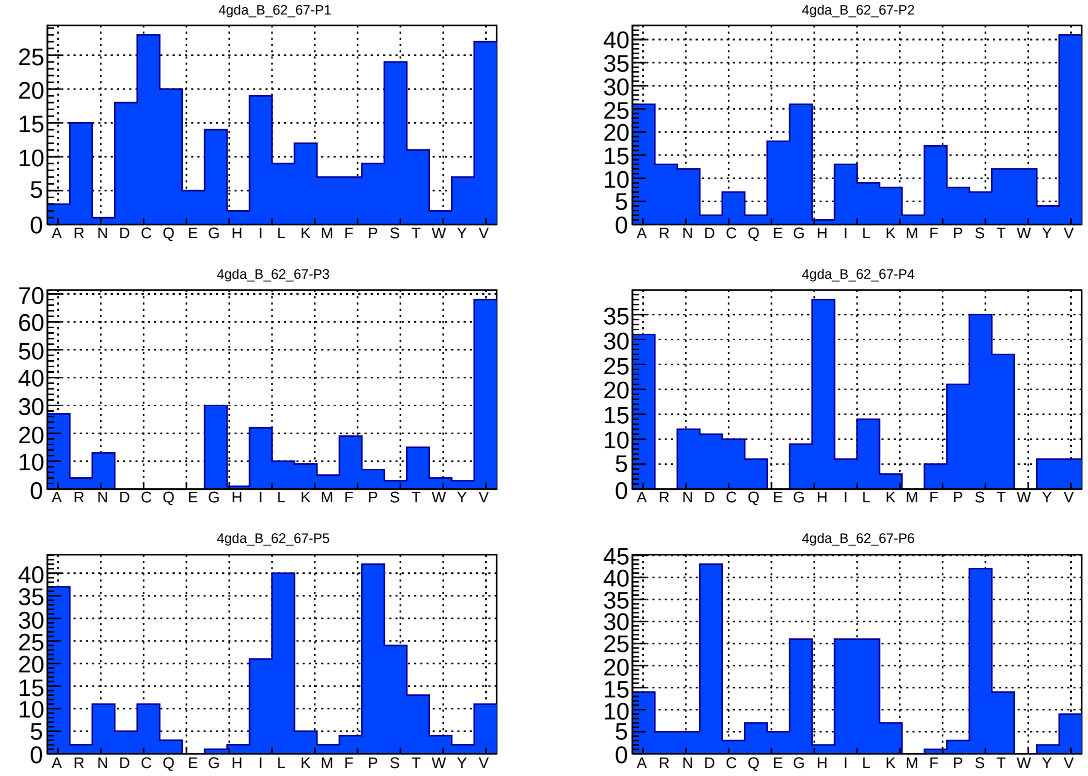

(xxii) cluster 22

Figure S3

Continued

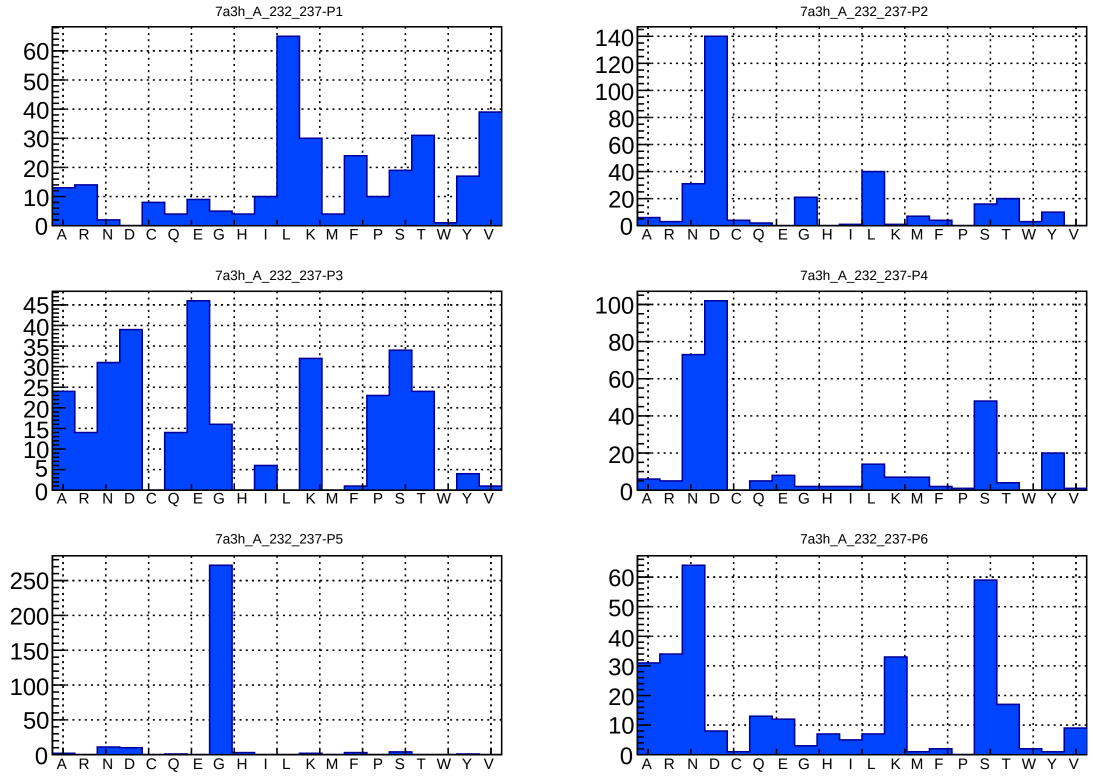

(xxiii) cluster 23

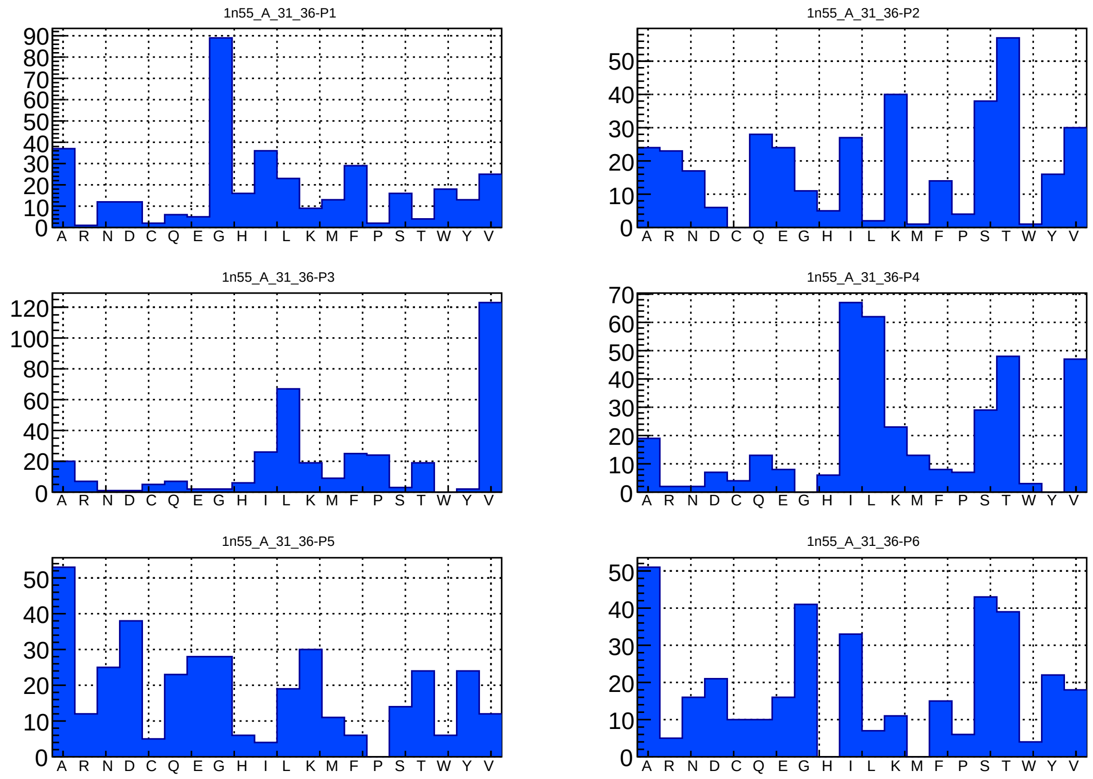

(xxiv) cluster 24

Figure S3

Continued

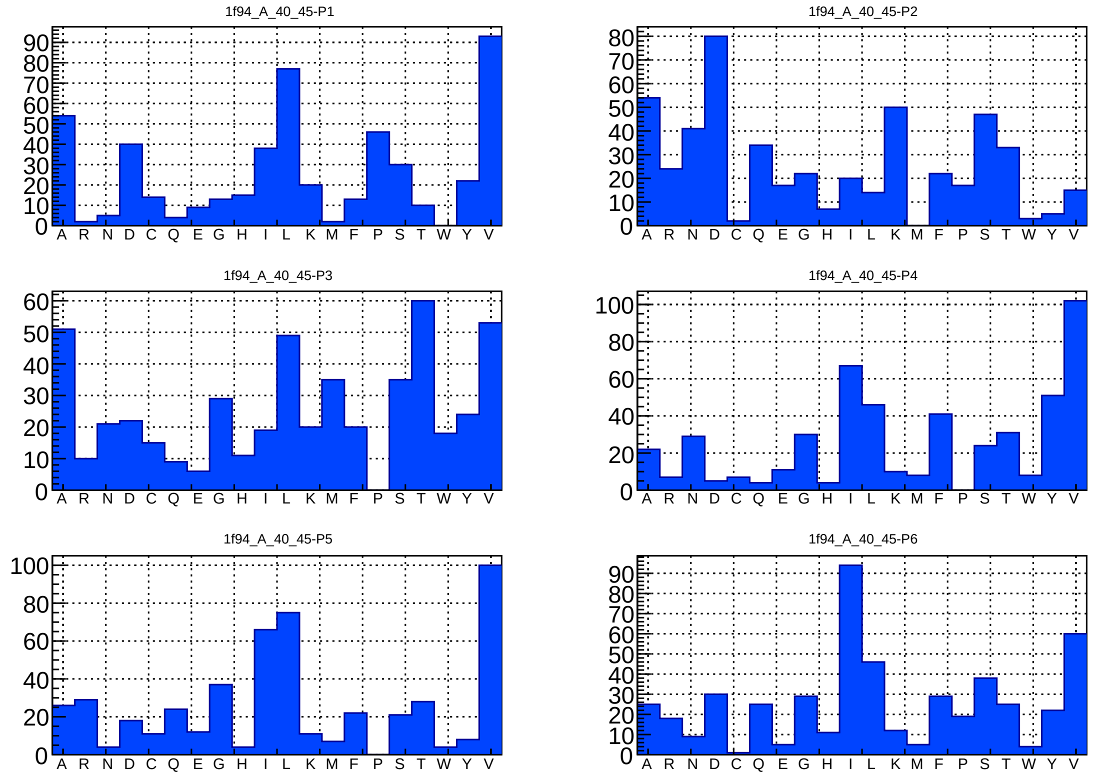

(xxv) cluster 25

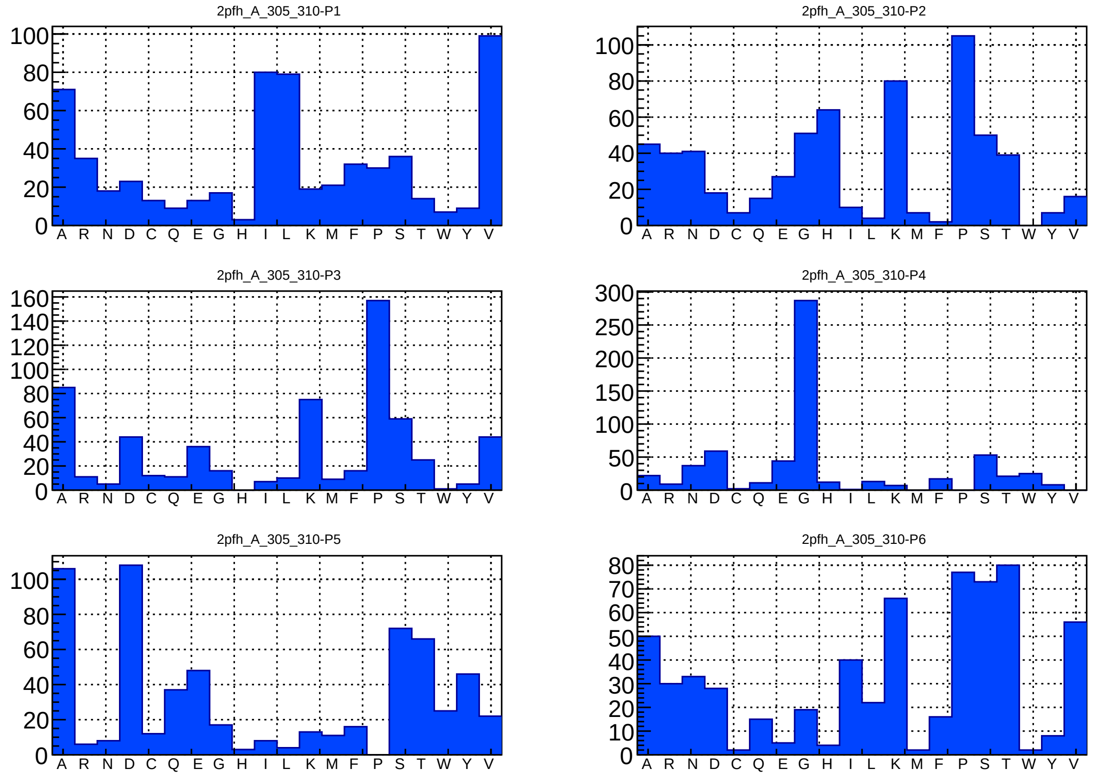

(xxvi) cluster 26

Figure S3

Continued

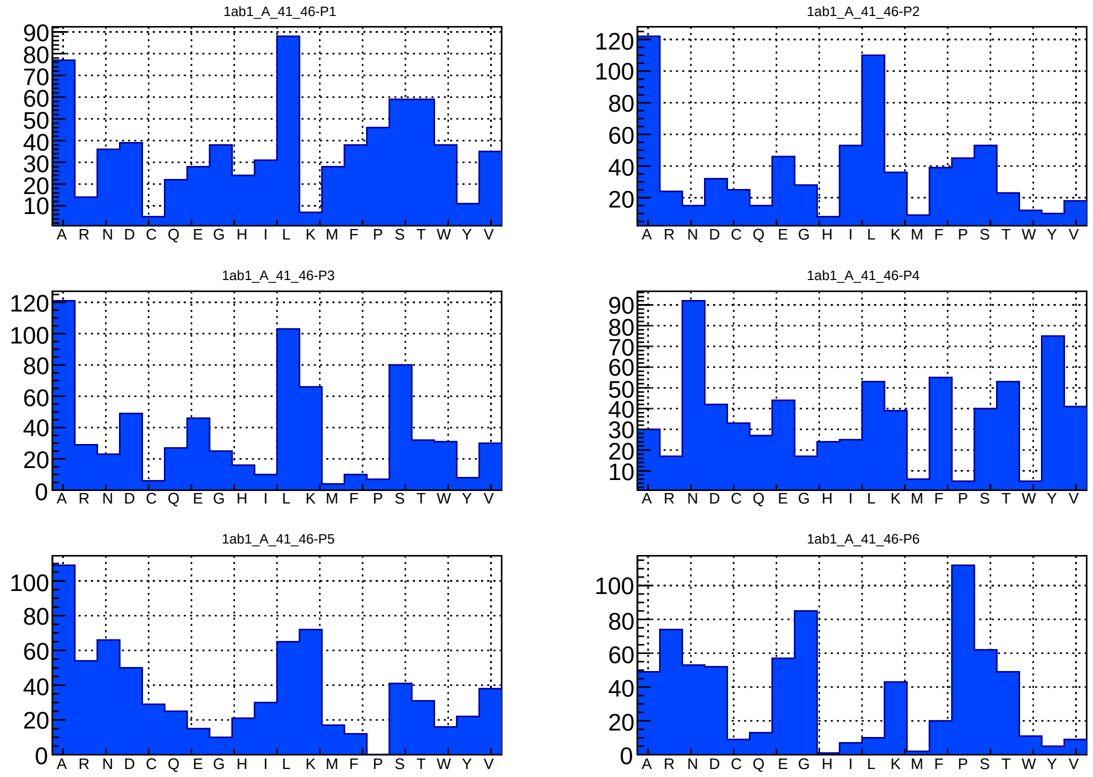

(xxvii) cluster 27

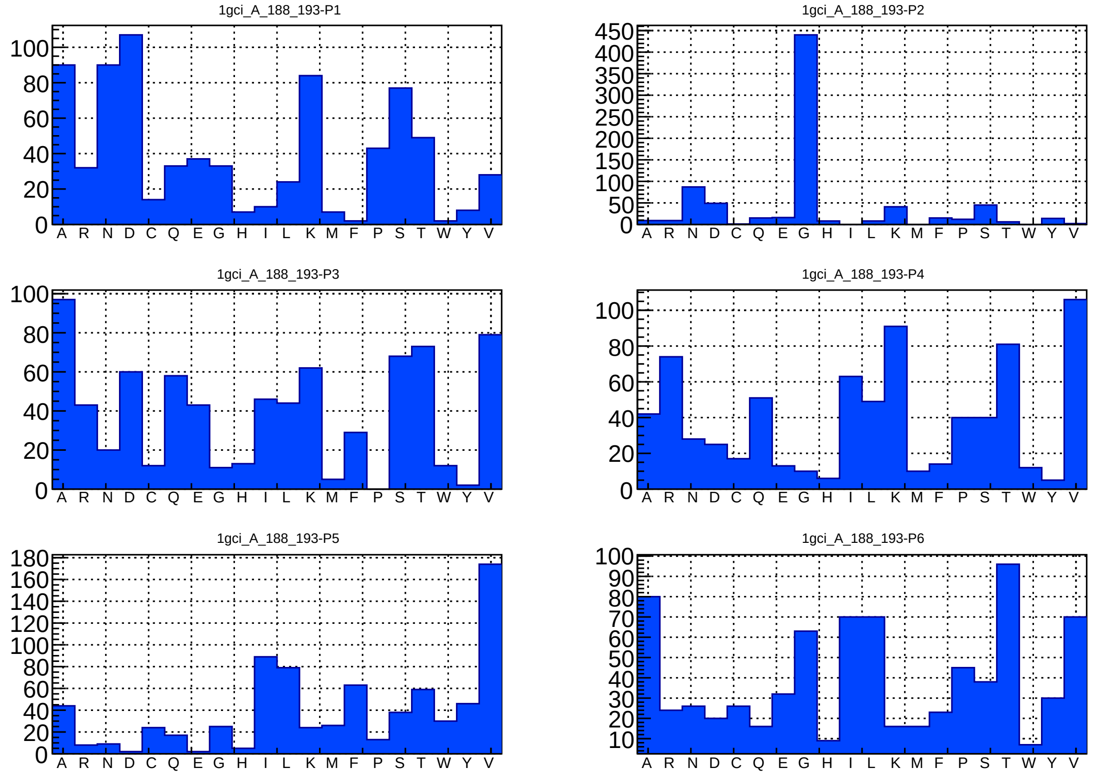

(xxviii) cluster 28

Figure S3

Continued

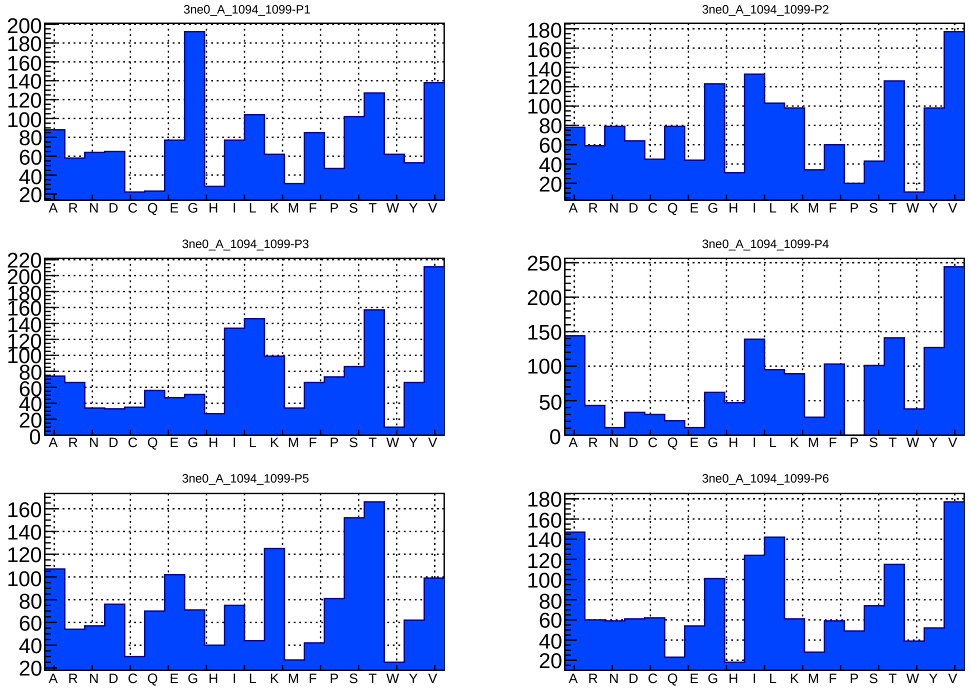

(xxix) cluster 29

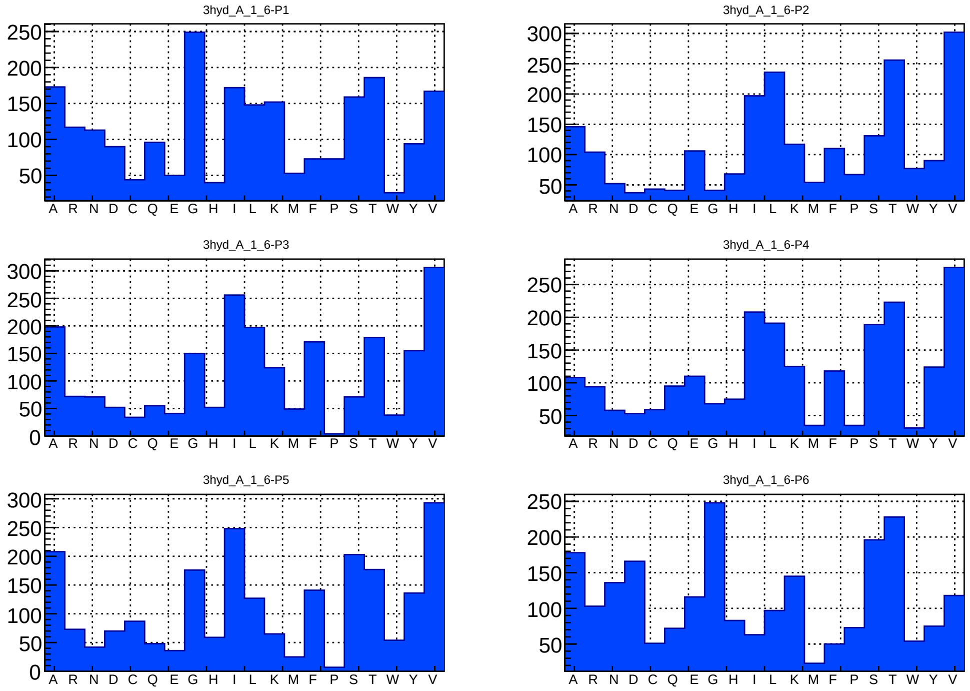

(xxx) cluster 30

Figure S3: Amino acid distributions of the 30 big clusters, which is obtained by elongation of the LLLLL subset of the cluster with initiator 1p1x A (80-84). The notations are the same as in Figure S2. Again, there are clear patterns in distribution of the amino acids especailly for the middle site P3 and P4 of the segment in each cluster.

Sequence	Cluster Initiator	Entry
CCATNV	1vyr_A (174-179)	1r2m_A (13-18), 1r2m_B (13-18), 2b97_A (13-18), 2b97_B (13-18)
KIGGQL	1gkm_A (163-168)	2hs1_A (14-19)
LKGDKL	1gkm_A (163-168)	1ix9_A (133-138), 1ix9_B (133-138), 1ixb_A (133-138)
ANVSAA	4f18_A (1244-1249)	4f1u_A (1244-1249)
DAFSRV	4f18_A (1244-1249)	1xg0_C (3-8)
IDQLDN	4f18_A (1244-1249)	3g46_A (81-86)
IMGERL	4f18_A (1244-1249)	2gg2_A (120-125)
LAVREQ	4f18_A (1244-1249)	1mwq_B (18-23)
LSCTSH	4f18_A (1244-1249)	1us0_A (301-306), 2i16_A (302-307), 2i17_A (302-307), 2pfh_A (301-306), 2pzn_A (301-306), 2qxw_A (301-306), 3ghr_A (301-306), 3ghs_A (301-306), 3lz5_A (301-306), 3m4h_A (301-306), 4gcA_A (301-306)
NIIGNV	4f18_A (1244-1249)	3soj_B (38-43)
STHKAV	4f18_A (1244-1249)	1j0p_A (23-28)
FVTRVQ	1cex_A (140-145)	2h3l_B (1355-1360)
NLKYLF	1cex_A (140-145)	4g78_A (127-132)
TKLGEV	1cex_A (140-145)	3aks_A (103-108)
CCNPAC	1a6m_A (56-61)	1hje_A (2-7)
MANDKL	1a6m_A (56-61)	3g46_A (61-66)
DAGMRF	1iee_A (47-52)	3odv_A (20-25)
ESGLSK	1iee_A (47-52)	1g2y_B (18-23)
ESGNVV	1iee_A (47-52)	2agt_A (126-131)
KDGVAD	1iee_A (47-52)	4a7u_A (91-96)
QQGLTL	1iee_A (47-52)	3akq_A (161-166), 3akt_A (161-166), 3akt_B (161-166)
SDGNGM	1iee_A (47-52)	1iee_A (100-105), 2vb1_A (100-105), 4b4e_A (100-105), 4lzt_A (100-105)
TDGSTD	1iee_A (47-52)	2vb1_A (47-52), 3lzt_A (47-52), 4lzt_A (47-52)
AGALGV	1brf_A (5-10)	1o7j_A (31-36), 1o7j_B (31-36), 1o7j_C (31-36), 1o7j_D (31-36)
DVANGD	1brf_A (5-10)	3ago_A (29-34)
EKSSGG	1brf_A (5-10)	2zq7_A (37-42), 2zqa_A (37-42)
GALDDV	1brf_A (5-10)	3agn_A (25-30)
IAAVNA	1brf_A (5-10)	3s6e_A (485-490), 3s6e_B (485-490)
IVSDGN	1brf_A (5-10)	1iee_A (98-103), 4b4e_A (98-103)
LAELGA	1brf_A (5-10)	2zq7_A (119-124), 2zqa_A (119-124)
SEYCCT	1brf_A (5-10)	2vu6_A (155-160), 3vhg_A (155-160), 3vjq_A (155-160)
TAADGL	1brf_A (5-10)	1mnz_A (342-347), 2glk_A (342-347), 3u3h_A (342-347), 4a8i_A (342-347)
AAAGAA	1ixh_A (200-205)	3zoj_A (143-148)
DESGNV	1ixh_A (200-205)	2agt_A (125-130), 2pzn_A (125-130)
KICGYI	1ixh_A (200-205)	1yk4_A (7-12), 2pya_A (7-12)
KSSGGR	1ixh_A (200-205)	2zq7_A (38-43), 2zqa_A (38-43)
KVLVDN	1ixh_A (200-205)	1vl9_A (62-67)
LESGLS	1ixh_A (200-205)	1g2y_B (17-22)
TILKRW	1ixh_A (200-205)	3aj4_A (14-19)
TKDGGY	1ixh_A (200-205)	1mxt_A (256-261), 1n1p_A (256-261), 1n4u_A (256-261), 1n4v_A (256-261), 1n4w_A (256-261), 2gew_A (256-261), 3cnj_A (256-261), 3gyi_A (256-261), 3gyj_A (256-261)

Table S1

Continued

ADGAIV	2o7a_A (62-67)	2gba_A (17-22), 2idq_A (17-22), 2ids_A (17-22), 2idu_A (17-22)
EQALGQ	2o7a_A (62-67)	3rq9_A (56-61)
FANLPV	2o7a_A (62-67)	1ix9_A (48-53), 1ixb_A (48-53)
FINIEH	2o7a_A (62-67)	2h3l_B (1383-1388)
FPTEDP	2o7a_A (62-67)	3f1l_B (206-211)
FRSVPT	2o7a_A (62-67)	4kxv_A (437-442)
KDCMLQ	2o7a_A (62-67)	3aj4_B (73-78)
LAKMDD	2o7a_A (62-67)	1nwz_A (15-20), 3pyp_A (15-20)
LDSCKV	2o7a_A (62-67)	1g4i_A (58-63)
LLKAKG	2o7a_A (62-67)	2nx0_A (71-76), 3qm5_A (71-76), 3qm6_A (71-76), 3qm7_A (71-76), 3qm8_A (71-76), 3qm9_A (71-76)
MEAQPQ	2o7a_A (62-67)	3e6z_X (9-14)
NFNTTM	2o7a_A (62-67)	1nqj_A (914-919)
PAGWFI	2o7a_A (62-67)	1n9b_A (226-231)
SSENNA	2o7a_A (62-67)	1vl9_A (85-90)
TEEEVG	2o7a_A (62-67)	3s6e_A (436-441)
DVKKRR	1gkm_A (9-14)	1mn8_C (29-34), 1mn8_D (29-34)
EVADGA	1gkm_A (9-14)	2idu_A (15-20)
EVSAVS	1gkm_A (9-14)	4hen_C (235-240)
GIAQAD	1gkm_A (9-14)	2e4t_A (371-376)
GTPEAL	1gkm_A (9-14)	3nir_A (20-25)
HGHPDQ	1gkm_A (9-14)	1mn8_A (74-79), 1mn8_B (74-79)
IEEPAS	1gkm_A (9-14)	3rq9_A (72-77)
IVNRTQ	1gkm_A (9-14)	4hen_A (217-222), 4hen_B (217-222)
KMDDGQ	1gkm_A (9-14)	1ot6_A (17-22), 1ot9_A (17-22)
KPTIMG	1gkm_A (9-14)	2ggc_A (117-122)
MVSKLS	1gkm_A (9-14)	1g2y_A (1-6)
NDDPQN	1gkm_A (9-14)	3rq9_B (28-33)
PLDESG	1gkm_A (9-14)	3u2c_A (123-128)
RQSTIL	1gkm_A (9-14)	3aj4_A (11-16)
RTMKDG	1gkm_A (9-14)	2ggc_A (215-220)
STWYNG	1gkm_A (9-14)	2ixt_B (236-241)
TCSSEN	1gkm_A (9-14)	1vl9_A (83-88)
TEDPQK	1gkm_A (9-14)	3f1l_B (208-213)
TTTPRA	1gkm_A (9-14)	1vyr_A (145-150), 2abb_A (145-150), 3p8j_A (145-150)
YVDKNS	1gkm_A (9-14)	3s6e_B (464-469)
YWNTDD	1gkm_A (9-14)	3hgp_A (93-98)

Table S1: Sequences that appear both in the 12 clusters and in protein structures which are not contained in the clusters before percolation. With the percolation from these new entries appearing in the third column, we can extend the loop clusters by 3%.
